# Function of human pluripotent stem cell-derived photoreceptor progenitors in blind mice

**DOI:** 10.1038/srep29784

**Published:** 2016-07-13

**Authors:** Alona O. Barnea-Cramer, Wei Wang, Shi-Jiang Lu, Mandeep S. Singh, Chenmei Luo, Hongguang Huo, Michelle E. McClements, Alun R. Barnard, Robert E. MacLaren, Robert Lanza

**Affiliations:** 1 Nuffield Laboratory of Ophthalmology, University of Oxford, Oxford, England; 2 Astellas Institute for Regenerative Medicine, 33 Locke Dr, Marlborough, MA 01752, USA; 3 Moorfields Eye Hospital NHS Foundation Trust NIHR Biomedical Research Centre, London, England; 4 Oxford University Hospitals NHS Trust Biomedical Research Centre, Oxford, England.

## Abstract

Photoreceptor degeneration due to retinitis pigmentosa (RP) is a primary cause of inherited retinal blindness. Photoreceptor cell-replacement may hold the potential for repair in a completely degenerate retina by reinstating light sensitive cells to form connections that relay information to downstream retinal layers. This study assessed the therapeutic potential of photoreceptor progenitors derived from human embryonic and induced pluripotent stem cells (ESCs and iPSCs) using a protocol that is suitable for future clinical trials. ESCs and iPSCs were cultured in four specific stages under defined conditions, resulting in generation of a near-homogeneous population of photoreceptor-like progenitors. Following transplantation into mice with end-stage retinal degeneration, these cells differentiated into photoreceptors and formed a cell layer connected with host retinal neurons. Visual function was partially restored in treated animals, as evidenced by two visual behavioral tests. Furthermore, the magnitude of functional improvement was positively correlated with the number of engrafted cells. Similar efficacy was observed using either ESCs or iPSCs as source material. These data validate the potential of human pluripotent stem cells for photoreceptor replacement therapies aimed at photoreceptor regeneration in retinal disease.

Rod and cone photoreceptors, which comprise the retinal outer nuclear layer (ONL), are the light sensing cells of the eye. They convert light signals into electrical impulses, initiating the visual transduction cascade which sends visual information to the brain. Mammalian photoreceptors do not have the capacity to regenerate, and when lost due to injury or disease, light is no longer perceived.

At present, there is no treatment to regenerate lost photoreceptors, and retinal degenerations account for most untreatable forms of visual impairment and blindness in the developed world. Retinitis pigmentosa (RP) is an umbrella term for a group of hereditary retinal degenerations which are characterized by an initial degeneration of rod photoreceptors followed by gradual loss of cones^1^, and remains one of the leading causes of untreatable blindness.

Cell replacement may provide a promising therapy for patients who have lost all photoreceptor cells due to degeneration. Indeed, pre-clinical studies in animals have shown improvement of visual function following transplantation of post-mitotic photoreceptor precursor cells in animal models with a varied range of retinal dysfunction^2^,^3^,^4^,^5^,^6^, including demonstration that transplanted post-mitotic mouse photoreceptor precursors are able to construct a new ONL and restored some visual function in completely blind mice^4^. However, for clinical application, post mitotic human photoreceptor precursors do not represent a suitable source of cells for cell replacement, as they develop only in the second trimester of pregnancy^7^. In order to obtain an expandable source of cells for transplantation, *in vitro* differentiation of human pluripotent stem cells (PSC) may be directed to obtain retinal tissue, and specifically photoreceptor precursors for the treatment of RP.

The first clinical trials using human PSC to treat vision loss commenced in 2011^8^. Human embryonic stem cell (hESC)-derived retinal pigment epithelium (RPE) cells were transplanted into patients suffering from macular degeneration. Medium- to long-term safety, graft survival, and possible biological activity of hESC-RPE in individuals with dry-age related macular degeneration (AMD) and Stargardt disease were recently reported^9^,^10^. Similarly, a clinical study using human induced pluripotent stem cell (iPSC)-derived RPE cells to treat wet-AMD patients was initiated in 2014. The goal of these clinical trials was primarily to assess safety, and in the long term to prevent the loss of photoreceptors by transplantation of RPE cells. However, photoreceptor transplantation for replacement of lost photoreceptors in forms of RP is not yet underway. There is a critical need for an efficient methodology to generate homogeneous populations of clinical grade human photoreceptor precursor cells, as well as an assessment of whether such cells can restore function in the completely degenerate retina.

Accordingly, photoreceptors derived from animal and human ESC or iPSC^6^,^11^,^12^,^13^,^14^,^15^,^16^,^17^,^18^,^19^,^20^,^21^,^22^,^23^,^24^,^25^,^26^,^27^,^28^ have been generated as candidates for disease modeling and photoreceptor cell replacement therapy. Pre-clinical studies suggest that PSC-derived photoreceptors may engraft and express rod photoreceptor markers in a remaining host ONL after transplantation^6^,^14^,^15^,^29^,^30^,^31^,^32^. However, to date there are no reports of successful transplantation of PSC-derived photoreceptors in animal models of widespread degeneration in which the host ONL is absent, which are most clinically relevant for cell replacement therapy in patient with end stage RP.

Furthermore, previously reported methodologies generate mixed populations of retinal cells, and thus involve either transplantation of mixed retinal cells, without selection for photoreceptors^6^ or alternatively required further purification steps, such as transduction of photoreceptor cells by a fluorescent marker, followed by fluorescence activated cell sorting (FACS). The later method critically impairs cell survival^14^ and is undesirable for human therapy. Alternative photoreceptor purification methods include magnetic-activated cell sorting (MACS), selecting rod photoreceptors by the cell surface antigen CD73 and other surface markers which have proven efficient for the enrichment of murine photoreceptor progenitors^33^,^34^,^35^,^36^,^37^, though extrapolation to human cells remains unproven.

The objective of the current study was to develop a clinically-adaptable method of providing pure, renewable populations of photoreceptor progenitors (PhRPs) appropriate for research and therapy. Here we describe a defined method for differentiation of human pluripotent stem cells (hPSC) into PhRPs, successfully using both human ESC and iPSC lines as starting materials. This synchronized differentiation process generates PhRPs homogeneously expressing photoreceptor-specific genes, which are able to further mature *in vitro* and form rod photoreceptor-like cells. We subreinally transplanted human PSC derived-PhRPs in mice with severe end-stage ONL degeneration and show that the human donor cells were able to survive and mature *in vivo* in the absence of a host ONL, and interact with residual retinal layers to generate partial visual function in formerly blind mice.

## Results

### Robust generation of retinal neural progenitors from human pluripotent stem cells under defined serum- and feeder-free condition

**Figure 1:**
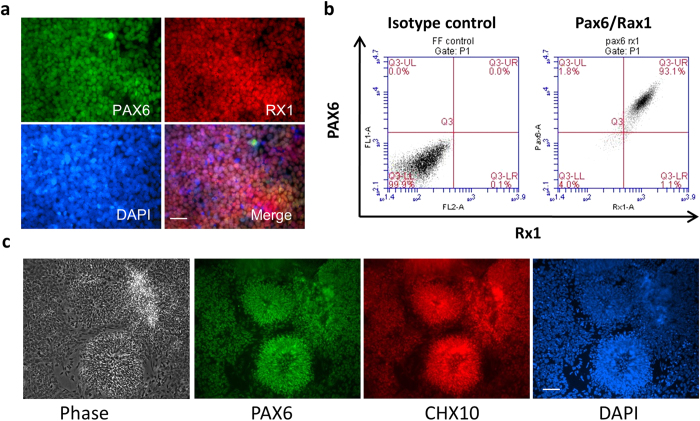
*In vitro* Differentiation of Human Embryonic Stem Cells towards Retinal Neural Progenitors. (**a**) Immunofluorescence staining shows co-expression of PAX6 and RX1 on day 13 eye field progenitors. (**b**) Quantification of PAX6 and RX1double positive eye field progenitors by flow cytometry analysis which shows >90% of them expressing both PAX6 and RX1 proteins. (**c**) Phase contrast image shows neural rosette structures of retinal neuronal progenitor cells (RNPC, far left) and immunofluorescence staining shows expression of PAX6 and CHX10 on RNPC at about day 30 after initial differentiation *in vitro*. Scale bar, 50 μm.

We developed a simple and efficient method to generate highly enriched retinal cells from human PSCs using a multi-step strategy (Supplementary Fig. S1). First, human PSCs were directly induced to differentiation in retinal induction medium (RIM) followed by exposure to neural differentiation medium (NDM) containing Noggin. After short exposure (usually 4 days) to RIM, cells at the edge of colonies became elongated and exhibited a columnar phenotype (Supplementary Fig. S2a), and small cells dominated the center of these colonies after further differentiation (Supplementary Fig. S2b). After 10 days of *in vitro* cell differentiation, more than 90% of cells expressed paired box 6 (PAX6), nestin and sex determining region Y-box 2 (SOX2) (Supplementary Fig. S2c,d), suggesting they were committed to neural stem cells. Approximately two weeks after differentiation and expansion in NDM, small cells dominated the whole culture with some large cells located at the edge of neural stem cell colonies (Supplementary Fig. S3a). Rapid upregulation of PAX6 and retinal homeobox gene 1 (RX1) transcription factors was observed, and the majority of them (>90%) were double positive for PAX6 and RX1 (Fig. 1a,b) at day 18. RT-PCR analyses showed expression of PAX6, RX1, LIM Homeobox 2 (LHX2), SIX homeobox 3 (SIX3), SIX homeobox 6 (SIX6) and T-Box 3 (TBX3) in these cells (Supplementary Fig. S3b). Real-time quantitative reverse transcription polymerase chain reaction (qRT-PCR) also demonstrated a dramatic decrease in the expression of octamer-binding transcription factor 4 (OCT4) and nanog, two critical pluripotent genes (Supplementary Fig. S3c). These results demonstrated a robust differentiation of hESCs towards retinal neural cells with gene profiles corresponding to eye field progenitors (EFP).

At day 19, EFP cells were collected and plated to form neural spheres in a suspension culture. After 3 days, neural spheres were re-plated on matrigel coated surface. Neural spheres were rapidly attached and cell bodies were spread out on the matrigel surface (Supplementary Fig. S4a) probably due to cell migration or active cell proliferation. Cells continually expanded and formed neural rosettes within a week (Fig. 1c (far left), Supplementary Fig. S4b). More than 95% of cells at this stage, including migrated-out neurons or neurons within aggregates, co-expressed PAX6 and Ceh-10 homeodomain containing homolog (CHX10) (Fig. 1c), suggesting they were retinal neural progenitor cells (RNPC). Recently published 3 dimensional (3D) retinal differentiation methods have demonstrated the formation of eyecup-like structures from human pluripotent stem cells. While this 3D method generates both retinal and forebrain neurons, the CHX10 positive RNPs are restricted to spheres with eyecup morphology. To check if our ESC-derived RNPs could form eyecup like structure in a suspension culture, we mechanically lifted RNPs from matrigel coated surface and plated them on an ultra-low attachment culture dish to form RNP spheres. Within 7 days, all spheres formed eyecup like structure (Figure S4c).

To validate the methodology, 6 hESC and 6 iPSC lines (Table S1), generated with different strategies, were subjected to retinal neuron differentiation and analyzed for the expression of PAX6 and RX1 by flow cytometry. Results demonstrated that all tested cell lines generated a near-homogenous population expressing these markers (Table S1), confirming the robustness of the retinal differentiation platform.

### Generation of photoreceptor-like progenitors and photoreceptor-like cells

**Figure 2:**
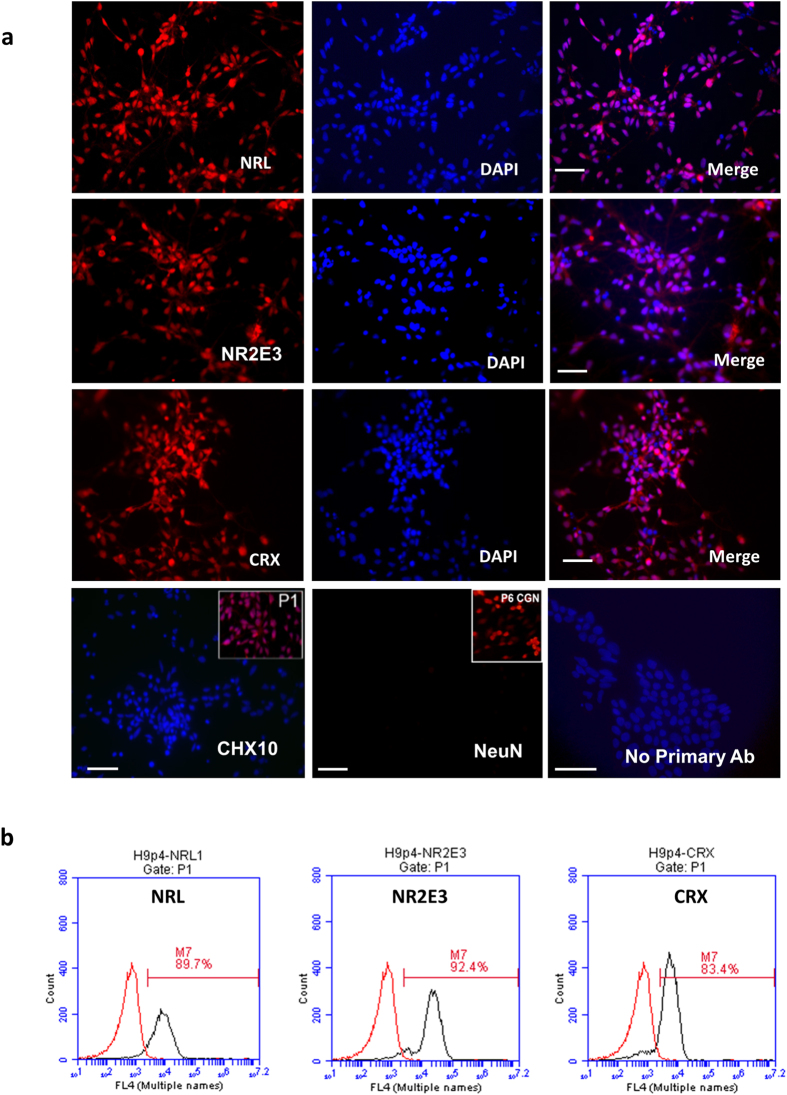
*In vitro* Differentiation of Retinal Neural Progenitors towards Photoreceptor-like Progenitors. (**a**) Immunofluorescence staining shows the expression of transcription factors NRL, NR2E3 and CRX in PhRPs at 90 –100 days after *in vitro* differentiation. CHX10 and neuN genes are negative in PhRPs at this stage; the upper right corner of the CHX10 image shows positive expression of CHX10 in RNPCs at day 30 (positive control); the upper right corner of the neuN image shows positive expression of neuN in mouse central nerve cells (positive control). 2^nd^ antibody only also shows negative staining. (**b**) Quatification of intracellular staining of NRL, NR2E3 and CRX as determined by Flow Cytometry analyses. Scale bar, 50 μm.

We next tested whether PSC-RNPCs were able to further differentiate into more mature retinal neurons such as photoreceptor progenitors (PhRP) and photoreceptors. After culturing H9-RNPCs in NDM without noggin supplementation, cells further differentiated into photoreceptor-like progenitors, as characterized by the expression of transcription factors involved in photoreceptor development. After four passage (approximately 3 months in NDM without noggin), dramatic morphological changes were observed (Supplementary Fig. S5a). Although neurons formed short neurites at this stage, neurite branching from the cell body was reduced or absent. Cells at this stage lost expression of CHX10, but were stained positive for Cone-Rod homeobox (CRX), neural retina leucine zipper (NRL), and nuclear receptor subfamily 2, group E, member 3 (NR2E3), key transcription factors that are essential for photoreceptor fate determination and development (Fig. 2). Less than 10% of them are positive for Ki67, a cellular marker that is strictly associated with cell proliferation (Supplementary Fig. S5b). Both neuN antibody and secondary antibody alone showed negative stains in these cells (Fig. 2a, bottom). These results indicate that cells were differentiating towards retinal photoreceptors, probably at the stage of PhRPs. Near-homogenous PhRPs were similarly generated from a human iPSC line, which expressed rod specific transcription factor, NRL (Supplementary Fig. S5c). Approximately 100 fold increase of cell number was observed from PSCs to PhRPs in three experiments using both ESC and iPSC lines.

**Figure 3:**
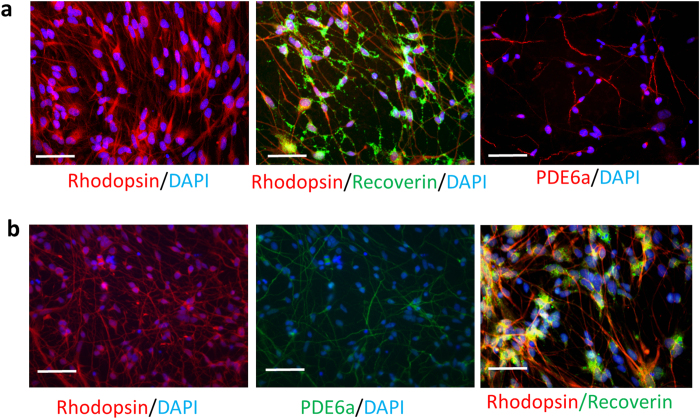
*In vitro* Generation of Mature Photoreceptor-like Cells from Human ESC/iPS-Derived PhRPs. Expression of rod photoreceptor markers, rhodopsin, recoverin and PDE6α in hESC (**a**) and iPSC-derived (**b**) photoreceptor-like cells two week after *in vitro* maturation. Scale bar, 50 μm.

To generate photoreceptor-like cells, PhRPs were further cultured in medium containing retinoic acid, DAPT, brain-derived neurotrophic factor (BDNF) and ciliary neurotrophic factor (CNTF). After two weeks, most cells (≈95%) became rod-like photoreceptor cells expressing rhodopsin, recoverin and phosphodiesterase 6 alpha (PDE6α) (Fig. 3a). Congruently, cells expressing photoreceptor-specific markers were also generated from iPS-PhRPs (Fig. 3b).

### Human PSC-derived PhRPs survived in the subretinal space of wild type mice

H9-ESC and HA-iPSC derived PhRPs were selected for further *in vivo* investigation due to their relevance for clinical application; the H9 ESC line is registered with NIH for use in academic collaboration and the HA-iPS line was generated using mRNA reprogramming technology^38^ which eliminates the risk of genomic integration and harmful alteration of the host genome. In order to discern transplanted human cells following transplantation, PhRP spheres were transduced by a recombinant serotype2 capsid-mutant adeno-associated virus (rAAV2/2 Y444F), expressing GFP under the photoreceptor-specific human rhodopsin kinase promoter. GFP expression was achieved *in vitro* 7 days post transduction in 70–90% of cells (Supplementary Fig. S6). To reduce culture time prior to transplantation, and validate the contained transduction of donor cells by AAV, PhRP derived from H9-ESC (ESC-PhRP) were first subretinally transplanted to WT mice (n = 4) 48 hours post transfection (before peak GFP expression). Transplanted human cells, positive for both GFP and human nuclear antigen (HNA), were observed in the subretinal space of WT mice 7 days post transplantation, without transduction of the host retina by GFP (Supplementary Fig. S7), confirming the contained expression of GFP in donor cells. Hence for transplantation in the severely degenerate retina, dissociated single PhRPs were transplanted 48 hours following AAV transduction.

### Human PSC-derived PhRPs survived in the severely degenerated subretinal space of *rd1* mice

*Rd1* mice were used as a host model of rapid and progressive RP with end-stage retinal degeneration. In these mice, retinal degeneration is caused by a null mutation in the rod photoreceptor cyclic GMP (cGMP) phosphodiesterase β subunit (Pde6b) gene^39^,^40^. Most rod photoreceptor cells are lost in the rd1 mouse by 3 weeks of age^41^, followed by progressive rod and cone photoreceptor degeneration over the first 8 weeks of life^42^ with absence of a functional ONL by 6–10 weeks of age^4^,^43^,^44^. At this late age, *rd1* mice represent a relevant model for studying transplantation in patients with end stage RP, as in *rd1* mice there is sever structural degeneration of the ONL, as appose to other retinal disease models such as the CRX knockout mouse, in which the retina is severely compromised functionally, but there is no structural degeneration.

Human PhRPs were transplanted into the subretinal space of *rd1* mice aged 10–12 weeks, at the end-stage of ONL degeneration (supplementary Fig. S8 shows the vast loss of ONL in the adult *rd1* by comparing WT and *rd1* retinal structures at 10 weeks). To prevent immune-rejection of human cells, immunosuppression with cyclosporine was administered to host mice^45^, starting 2 days prior to transplantation and continuing throughout the experiment.

A first group of *rd1* mice received transplantation of ESC-PhRPs, a second group received transplantation of iPSC-PhRPs (n = 8 per group, unilateral injections). A third group received transplantation of H9-ESC derived retinal neural progenitor cells (RNPCs)(n = 5) to control for PhRP cell survival. And a final group received a sham transplantation of PBS to control for the surgical manipulation (n = 8). All cells were transduced by rAAV2 Y444F. GFP prior to transplantation and animals in the RNPC transplantation group were investigated alongside PhRP groups to control for the undesired transfection of the host ONL by free AAV particles that could potentially be delivered with cells.

**Figure 4:**
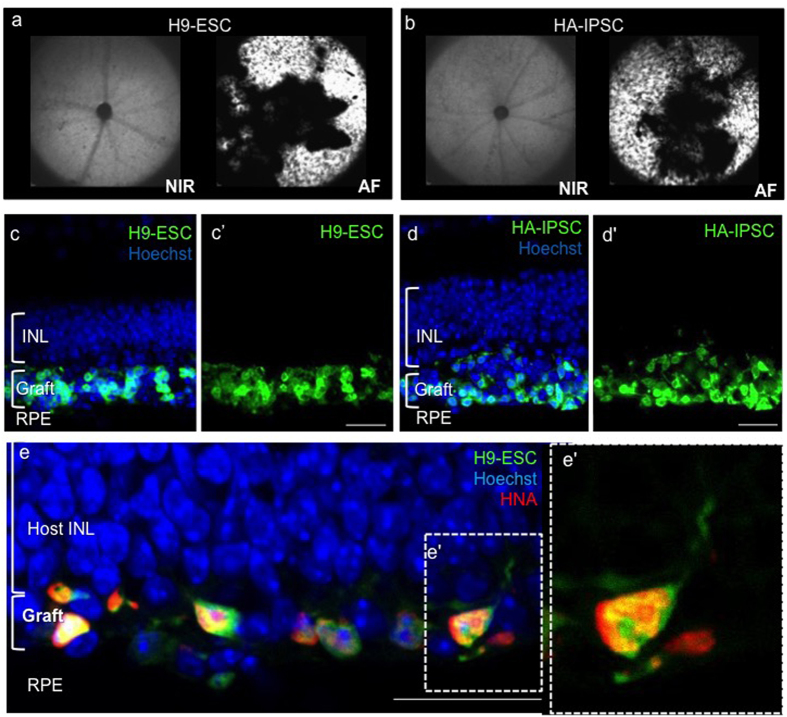
Transplanted Human ESC-PhRPs and iPSC-PhRPs Survive in the Subretinal Space of rd1 Mice. Scanning laser ophthalmoscopy (SLO) was performed *in vivo* three weeks post transplantation to assess the extent of surviving donor cells; GFP positive cells are observed in autofluorescence (AF) mode as white dots or clusters (black areas represent areas of retina which were not seeded with transplanted cells, due to incomplete detachment of the retina around the optic nerve head). Representative near-infrared (NIR) and AF fundus images of *rd1* mice show a homogeneous presence of GFP+ cells in the two treatment groups: ESC-PhRPs (**a**) and iPSC-PhRPs (**b**). Histological assessment 3 weeks post transplantation revealed ESC-PhRP (**c**-c’) and iPSC-PhRP (**d**-d’) derived cell layers (green) between the retinal pigment epithelium (RPE) and inner nuclear layer (INL) of the *rd1* retina, replacing the absent outer nuclear layer (ONL) in the adult *rd1* mice; (**e**-e’) GFP+ cells were stained with human nuclear antigen (HNA) which co-localized with GFP; indicating that the GFP signal observed *in vivo* in treated animals was indeed an indicator of transplanted human PhRPs. Scale bar, 25 μm.

Three weeks post transplantation a distinct layer of GFP+ cells was observed *in vivo* in animals injected with ESC-PhRPs (Fig. 4a) and iPSC-PhRPs (Fig. 4b). In the same mice, histology revealed GFP+ human cells residing in the subretinal space of animals in both ESC-PhRP (Fig. 4c,c’) and iPSC-PhRP (Fig. 4d,d’) treated groups. On average 6.45 ± 1.1% (mean ± 1 S.E.M) of transplanted ESC-PhRPs and 5.7 ± 1% of iPSC-PhRPs survived in the subretinal space of rd1 mice 3 weeks post transplantation, (n = 8 specimens each). Less than 0.001% of 2 × 10^5^ GFP-RNPCs, which were transplanted as a control for cell survival, were observed in the subretinal space of rd1 mice, and cells were only evident in eyes of 2 of 5 transplanted animals (see Supplementary Fig. S9). This validates the significance of appropriate developmental stage of PSC-derived retinal cells for survival following transplantation. The absence of GFP+ cells in the control group also further confirms that GFP+ cells observed in the PhRP-transplanted treatment groups were indeed donor-derived cells and not a result of host retina transduction.

Grafted GFP-positive cells expressed human nuclear antigen, and developed processes to interact with host circuitry. However, similar to a previous a report of cell transplantation in the severely degenerate adult *rd1* mouse^4^, transplanted PSC-PhRPs in this study did not adopt normal photoreceptor morphology and orientation (Fig. 4e,e’) as described when retinae with a residual ONL were treated with mouse rod progenitors^5^,^46^,^47^,^48^ or human PhRPs^6^.

### Transplanted human PSC-derived PhRPs mature *in vivo* in the severely degenerated retina

**Figure 5:**
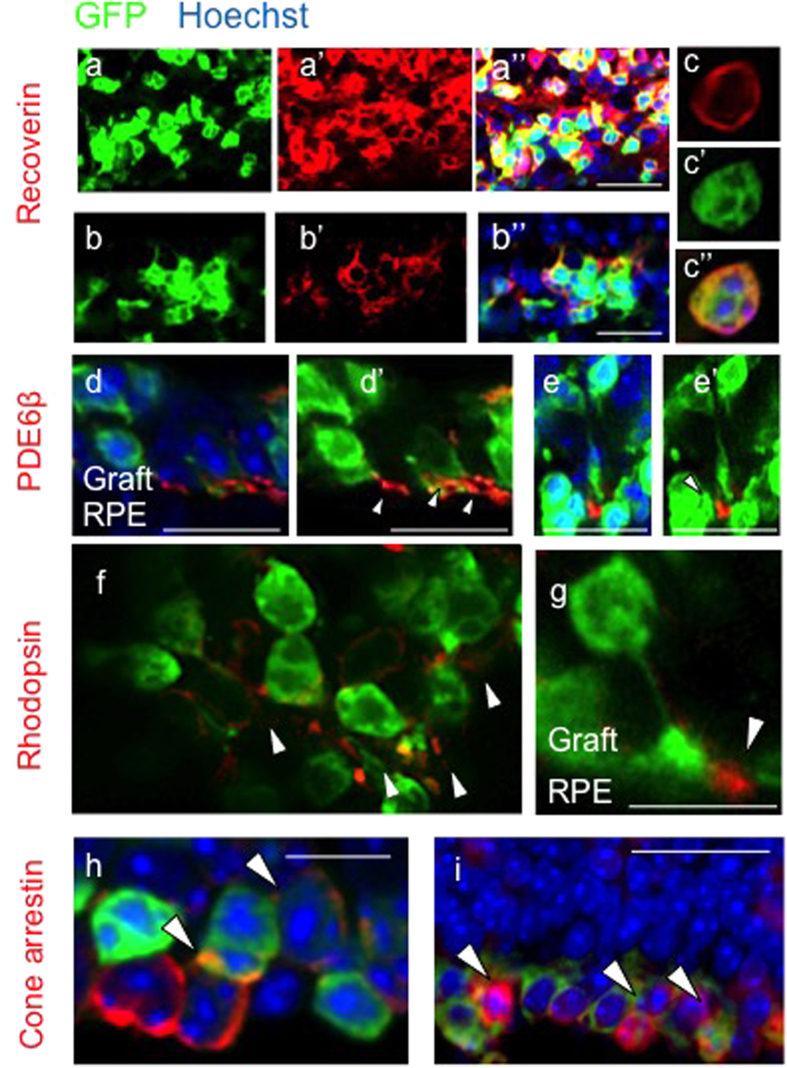
Transplanted Human ESC-PhRPs and iPSC-PhRPs Express Mature Photoreceptor Markers *in vivo*. Immunofluorescence staining 3 weeks post transplantation shows expression of mature photoreceptor markers in transplanted human PhRPs (green). In all images cells are located in the subretinal space and oriented so that the host INL is located at the top of the image and the RPE at the bottom. The pan-photoreceptor marker recoverin was observed within the reconstructed layer of cells in animals treated with both ESC-PhRPs (**a**) and iPSC-PhRPs (**b**,**c**). The rod specific enzyme phosphodiesterase β6 (PDE6b), which is necessary in phototransduction and is absent in rd1 mice due to mutation was reinstated in the retina and located in the outer processes of transplanted ESC-PhRPs (**d**-d’) and iPSC-PhRPs (**e**-e’). The rod specific protein rhodopsin, which is normally located in outer segment membrane disk was also observed in outer segments of ESC-PhRPs (**f**) and iPSC-PhRPs (**g**). Cone arrestin was observed in GFP+ cells, indicating that a subset of human cells matured to produce cone photoreceptors. Scale bar, 20 μm.

**Figure 6:**
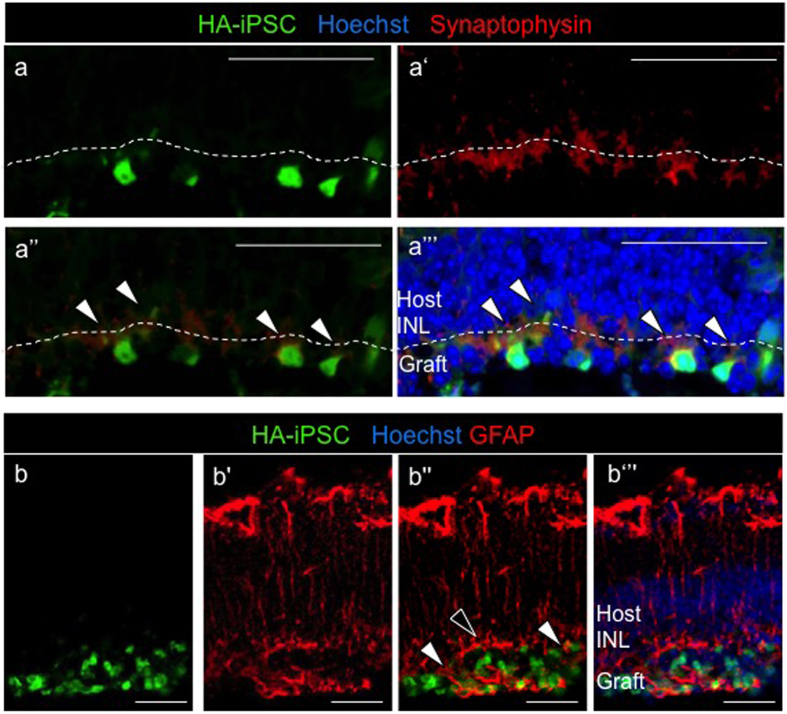
Graft-Host Connectivity. (**a**-a”’) Expression of synaptophysin, a synaptic marker, was present between the host INL and the GFP-positive graft. Synaptophysin is localized between the host and graft and is expressed in transplanted cells (white arrows) indicating synaptic transmission between human iPSC-derived grafted cells and the host rd1 retina; (**a**) GFP (human cells); (a’) synaptophysin; (a”) merged image of GFP and synaptophysin; and (a”’) merged image of GFP, synaptophysin and DAPI. The dashed line delineates the boundary between the host INL and the graft. (**b**-b”’) Glial fibrillary acidic protein (GFAP), a protein expressed by inner retinal astrocytes and activated Müller glia, is expressed by the glial cells of the host retina (red). Gliosis in the degenerate retina may occur to protect the retina from further damage, and a horizontal glial scar at the edge of the host ONL was observed in some areas of the retina (black arrow). However, glial processes were also observed to extend into the graft (green), without formation of a complete glial barrier between host and graft (white arrows). **(b**) GFP (human cells); (b’) GFAP+ host glial cells; (b”) merged image of ((**b**) GFP) and ((b’) GFAP); (b”’) merged image of GFP, GFAP and DAPI. Scale bar, 20 μm.

Three weeks post transplantation in the adult *rd1* eye, PSC-PhRP located in the subretinal niche, examined by immunohistochemistry, expressed mature photoreceptor proteins: The pan-photoreceptor marker recoverin was expressed by both ESC-PhRPs and iPSC-PhRPs (Fig. 5a–c, respectively). The rod-specific cGMP phosphodiesterase β6 (PDE6β) was observed in the outer processes of transplanted cells (Fig. 5d-d’); a deficit in this phototransduction-enzyme is the underlying cause of retinal degeneration in the *rd1* mouse, certifying its expression by donor rods. The rod specific protein rhodopsin was also expressed in transplanted cells of both treatment groups (Fig. 5f,g). Localization of rhodopsin and PDE6β proteins was correctly confined to the developing outer processes of cells, demonstrating maturation of human rod cells in the degenerate *rd1* subretinal niche. Cone arrestin protein was observed in GFP+ cells (Fig. 5h,i), indicating maturation of human cone photoreceptors within the transplanted photoreceptor cohort. Normal photoreceptors signaling can be identified by the synaptic vesicle glycoprotein, synaptophysin which is normally expressed in photoreceptor synaptic terminals in contact with bipolar cells. Three weeks post transplantation synaptophysin protein was present between the human graft and the host retina, indicated an interaction between donor cells and host circuitry (Fig. 6a,a”’). Gliosis is a limiting factor in CNS regeneration and has been proposed to represent a cellular attempt to protect remaining tissue from further damage^49^. Gliosis in the retina predominantly involves Müller glia cells, which upregulate the glial fibrillary acidic protein (GFAP) of the Müller cell processes at the edge of the neural retina^50^. The presence of a glial scar in the retina may stand as a physical barrier to cell integration^51^. Host Müller glia (GFAP positive) were found to form a glial barrier at some areas of the retina, however glial processes were also found to extend into the engrafted human PhRPs (Fig. 6b,b”’), and grafted cell extended processes into the host INL (see Supplementary Fig. S10), supporting a degree of integration between the host and graft layers without a significant glial barrier forming between them. Confocal stacks from a whole-retina flatmount demonstrated the morphology and formation of cell processes in transplanted PhRPs 3 weeks after transplantation into the rd1 subretinal space (see Supplementary Fig. S10).

### Recovery of basic visual function in animals with end-stage retinal degeneration

In order to further assess transplanted cell maturation and integration into host circuitry, we tested for changes in basic behavioral function in treated *rd1* mice. Behavioral testing was conducted after dark adaptation and using dim (~10 lux) 510 nm illumination which is close to the wavelength of light preferentially detected by rod cells. This light stimulus was selected in order to target transplanted rod cells while eliminating the contribution of potentially functional host cone cells or intrinsically-photosensitive retinal ganglion cells (ipRGCs).

**Figure 7:**
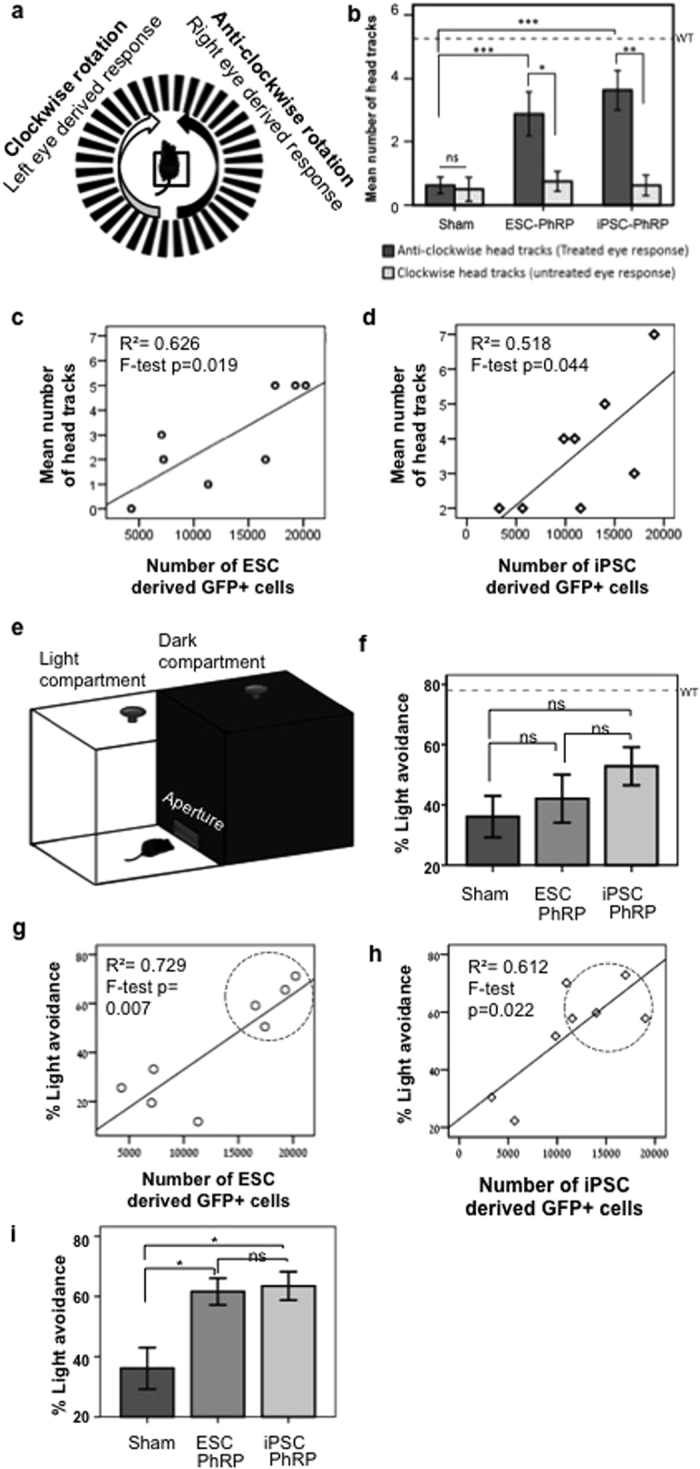
Recovery of Basic Visual Responses in rd1 Mice Following Transplantation of Human PhRPs Correlates to Number of Engrafted Cells. (**a**) Schematic of the optomotor response (OMR) test arena and expected response to the direction of drum rotation. (**b**) Mean OMR 3 weeks post-transplantation indicating an improvement in OMR driven by treated eyes (dark grey) compared to paired untreated eyes (light grey) after transplantation of ESC-PhRPs (paired sample t-test, t = 2.86, p = 0.024) and iPSC-PhRPs (paired sample t-test, t = 5.02, p = 0.002); In the sham treated group there were no differences in OMR driven by treated and untreated eyes (paired sample t test, t = 0.31, ns). Furthermore, OMR was improved in PhRP treatment groups compared to sham treatment (one way-ANOVA, F = 7.8, p = 0.003), with an increase in the response in both ESC-PhRP (p < 0.05) and iPSC-PhRP (p < 0.005) treated animals (Bonferroni test for multiple comparisons). (**c**) A positive correlation was observed between number of head tracks and number of GFP+ cells in animals treated with ESC-PhRPs (n = 8, R^2^ = 0.729, F = 16.13, p < 0.01) and (**d**) iPSC-PhRPs (n = 8, R^2^ = 0.612, F = 9.46, p < 0.05). (*p < 0.5, **p < 0.01). (**e**) Schematic of the light avoidance apparatus. (**f**) There were no differences between the three groups in mean light avoidance responses (F = 1.43, p = 0.261 [ns]). However, a positive correlation was observed between number of GFP+ cells and light avoidance behavior in individual animals of (**g**) ESC-PhRP (R^2^ = 0.729, F = 16.13, p < 0.01) and (**h**) iPSC-PhRP treated group (R^2^ = 0.612, F = 9.46, p < 0.05). (**i**) Comparing only animals with above-median numbers of GFP+ cells (encircled in (**g**,**h**)) a significant difference was observed between the three groups (X^2^ = 6 (df2), p < 0.05) showing improvement in ESC-PhRP treated (n = 4, p < 0.05) and iPSC-PhRP treated (n = 4, p < 0.05) subgroups. The dashed line in (**b**,**f**) represents the mean response of age-matched wild-type mice. Error bars represent ± S.E.M.

We assessed the presence of an optomotor response (OMR) to a rotating grating, by adapting a previous protocol^52^. The test animal is placed in the center of a rotating striped drum. An OMR is recorded when the animal turns its head to track the rotating grating. Tracking in each direction is independently driven by one eye^53^: an OMR elicited by rotation in a clockwise direction is driven by the left eye, and a response to anti-clockwise rotation is driven by the right eye (the treated eye in this study) (Fig. 7a). A significant increase in the number of direction-dependent head tracks was found when comparing head tracks driven by treated eyes compared to untreated eyes of animals transplanted with ESC-PhRPs (Paired t test, t = 2.86, p < 0.05) and iPSC-PhRPs (Paired t test, t = 5.02, p < 0.01), but not in the shame treatment group (paired t test, t = 0.31, ns) (Fig. 7b). A difference was also found between the three treatment groups in the number of treated eye-derived head tracks (ANOVA, F = 7.8, p < 0.01), with post hoc analysis revealing improvements in ESC-PhRP (p < 0.05) and iPSC-PhRP (p < 0.01) groups compared to sham treatment, but no difference between the two PhRP treatments (ns) (Fig. 7b).

We correlated behavioral performance to the number of positively identified human cells in each animal (Table S2), and found a positive correlation between the number of GFP+ human cells and performance in OMR for both ESC-PhRP (R^2^ = 0.626, F = 10.0, p < 0.05; Fig. 7c) and iPSC-PhRP (R^2^ = 0.518, F = 6.45, p < 0.05; Fig. 7d) treated animals.

To qualify further the observed behavior, we conducted a light avoidance assay, as previously described^4^. Mice were free to transition between light and dark compartments of a test arena, and the percent of time spent avoiding light was quantified (for schematic representation see Fig. 7e); As mice are nocturnal animals, they tend to avoid bright environments^54^, however, this is dependent on the perception of light, thus the tendency to avoid light is measured to infer visual function. Here, rd1 mice in the three treatment groups did not differ in their tendency to avoid light (ANOVA, F = 1.4, p = 0.261 (ns); Fig. 7f). Measures of anxiety-related behavior were examined in order to assess whether a difference in anxiety between the groups may contribute to the result, but no differences in anxiety-related behavior was observed (see Supplementary Fig. S11). Nevertheless, a trend was observed, in which some mice in the PSC-PhRP treated groups responded more than sham transplanted mice, hence in order to assess the reason for variability in the results, light avoidance behavior was qualified to the number of engrafted cells in each animal. As with the OMR, we found that number of engrafted cells in individual animals strongly correlated with light avoidance behavior in both ESC-PhRP (R^2^ = 0.729, F = 16.1, p < 0.01; Fig. 7g) and iPSC-PhRP (R^2^ = 0.612, F = 9.46, p < 0.05; Fig. 7h) treated animals (n = 8 per group) (individual results are displayed in Table S2). Since light avoidance behavior is driven by light-intensity, which might have a threshold effect to the number of light-sensitive cells, we performed a sub-analysis of the three groups, including only animals with above-median number of surviving cells (top 50%) in the treated groups. In this case, though it should be qualified that numbers were small in this sub-analysis, a difference was observed between treatment groups (χ^2^ = 6.0, p = 0.041) with an increase in light avoidance in both ESC-PhRP (n = 4, p < 0.05) and iPSC-PhRP (n = 4, p < 0.05) treated animals compared to sham treatment (Fig. 7i).

In order to exclude the possibility that the functional improvements presented here were due to neuroprotection of residual host cone-photoreceptors, remaining cones in treated rd1 mice were examined histologically using staining against cone-arrestin. As expected, some residual host cones were present in PSC-treated and sham rd1 mice^55^; however remaining cones were morphologically abnormal, with absence of inner and outer segments (see Supplementary Fig. S12). Furthermore, no difference was observed in the number of residual host-cones between ESC-PhRP, iPSC-PhRP or sham treatment groups (One way ANOVA, n = 5 per group, F = 0.56, p = 0.58, ns) (Supplementary Fig. S12).

## Discussion

Collectively, the data presented show that PhRP cells can be derived from both human ESC and iPSC lines to produce a source of cells suitable for photoreceptor cell replacements in a model of end stage RP. Transplanted PhRP cells survived in the completely degenerate retina of immunosuppressed mice with end-stage retinal degeneration and formed a new layer, replacing the degenerated ONL. They became immuno-positive for phototransduction proteins, and adopted morphology consistent with generating a primitive outer segment. The restoration of basic behavioral light responses correlated with the number of engrafted cells in the subretinal space, using both ESC and iPSC-derived PhRP.

A completely serum-free and feeder-free direct differentiation system efficiently supports the development of functional PhRPs from human PSCs. By synchronizing the differentiation process, this simplified system can be used to reproducibly generate highly enriched retinal PhRPs on a large scale from human PSC lines. This system is also amenable to the development of an *in vitro* GMP-compliant retinal cell manufacturing protocol for future preclinical and human studies. In particular, this differentiation method allows generation of retinal neurons at different development stages, which could be used to target various retinal degeneration diseases at different progression stages. In the developing vertebrate retina, diverse neuronal subtypes are generated from multipotent RPCs follows a stereotyped sequence^56^, and many photoreceptor specific transcription factors, such as NRL, CRX and NR2E3 are expressed during final mitosis in retinal progenitors and in early post-mitotic precursors^57^, which is consistent with our observation. NRL is a transcription factor which regulates the expression of rod photoreceptor specific genes including rhodopsin. NR2E3 is a suppressor of S-cone in the human retina^58^, and co-expression of NR2E3 and NRL may drive cell differentiation to produce rod photoreceptor-like cells.

In addition to efficacy, safety is an ultimate concern in transplantation of derivatives from PSCs. Human iPSC used for transplantation in this study were generated by a non-integrative mRNA approach, so addressing the major limitation of clinical application of iPSCs generated from integrative delivery systems. Additionally, current approaches to photoreceptor differentiation generate a mixture of retinal neurons requiring further purification steps to obtain pure photoreceptor progenitors as unselected mixed population of donor cells are associated with high risk of safety concerns and low transplant efficiency. In comparison to previously published embryoid body (EB)-based 3D cell differentiation methods, we initiated direct differentiation of human ESC and iPSC toward retinal photoreceptor cells at an extremely low seeding density on the matrigel surface. Supplied with a single BMP inhibitor in the differentiation medium, our method selectively generated PAX6 and CHX10 positive RNPCs, leading to further differentiation into PhRPs and photoreceptor-like cells that homogeneously express cell-specific genes without further cell sorting. Thus, our method provides an important step towards manufacturing high quality human photoreceptor cells from PSCs.

This new method generated a near homogenous population of PhRP (>90%) expressing markers specific for photoreceptor cell fate with less than 10% of cells expressing the cell proliferation marker Ki67. On further culture these cells were able to generate a population of cells in which over 95% of cells express photoreceptor specific markers. The homogeneous population used in this study is comparable to the population of cells attained following FACS sorting in previous studies using human ESC-derived PhRP^73^, where cell sorting yielded a cell suspension in which 90% of cells could subsequently be histologically labeled as rod or cone photoreceptors. Correspondingly, in another study^31^, after transplantation of mouse iPSC, the proliferation marker Ki67 was still detected in cells three weeks following transplantation, although rigorous MACS cell purification was applied for depletion of the pluripotency marker SSEA1, and no tumor formation was detected.

The human PSC-derived PhRPs used in this study were not further sorted after cell differentiation, and matured to restore a degree of vision to blind mice with no obvious adverse effect three weeks post transplantation. These results provide support for the use of this synchronized differentiation approach for production of human PhRP for transplantation, although further investigational new drug (IND)-enabling studies will be required to assess the safety of these cells prior to application in a clinical setting.

Human ESC and iPSC derived PhRPs were subretinally transplanted in the *rd1* mouse model of end-stage RP. We transplanted PhRPs in this rapid degeneration model at 10–12 weeks of age, a time point in which there is no remaining ONL^4^,^43^. Robust cell survival and *in vivo* expression of photoreceptor markers by transplanted cells three weeks post transplantation suggest that the severely damaged retina is a permissive space for human cell maturation. Further functional and histological characterization of the mechanisms at the base of visual repair by these cells as well as assessment of transplanted human cells beyond three weeks of transplantation will be needed in order to evaluate the full maturation and connectivity of human photoreceptors, which may include more complete OS formation and OS-specific protein expression over time. A further challenge to cell replacement is graft-host connectivity. Glial scarring and the host outer limiting membrane (OLM) are two features that may cause physical barriers to donor-host connectivity. In the rd1 mouse, OLM integrity is disturbed with aging, however, glial scaring increases^2^. We therefore studied glial processes in host *rd1* animals and excluded the formation of a complete glial barrier between donor cells and host INL. Survival, maturation and connectivity in the completely degenerate retina has been achieved by transplantation of mouse primary cells in suspension^4^ or mouse PSC-derived 3D retinal sheets^43^, but has not been shown before using human derived photoreceptor cells.

In the current study we have shown that transplanted human cells formed a distinct layer in the *rd1* subretinal space, resembling in some ways the configuration of the subretinal electronic retina^59^,^60^ in human clinical trials for end-stage RP. The lack of differentiation into vertical photoreceptor profiles with long outer segments is in keeping with observations from transplantation experiments in the *rd1* mouse host and may result from the inability for transplanted suspension-cells to become radially orientated to the RPE in the compressed outer nuclear environment in end stage degeneration^4^,^29^,^30^. The transplantation of retinal sheets rather than suspension cells may increase appropriate cell configuration. While there have been previous reports of visual function arising from very few cells, the abnormal shape of cells in this study would most likely reduce the light sensitivity of these photoreceptors; however, the large number of surviving cells observed with SLO and histologically in some animals would largely compensate for reduced sensitivity of individual cells. Similar to the subretinal electronic retina, electrical current changes generated by the transplanted cells may influence bipolar and horizontal cells even without conventional synapses, if the cells are in close enough apposition. The positive results with the subretinal implant which has 1,500 pixels^59^ demonstrate future potential of a cell therapy approach, which could implant millions of cells^61^. While some light sensitivity has been previously shown to be possible without the elaboration of outer segments in transplanted human cells^6^ and photoreceptors with profound structural damage have been shown to support vision in mice^62^, the abnormal cell morphology and lack of radially orientated cells in our study led us to consider an alternative explanation of neuroprotection of residual host cones.

Neuroprotection and photoreceptor rescue by transplanted RPE cells has been shown in the *rd1* mouse^63^, however, this was achieved in 2 weeks old mice, a stage in which there were still remaining photoreceptors in the ONL, making rescue by neurotrophic factors more likely. At 10–12 weeks of age, a small number of cones exist in the peripheral area of retina^64^; however, the number of remaining photoreceptor cells in *rd1* mice would not be sufficient to account for visual restoration. Nonetheless, if the existing cones are in a dormant or unhealthy status, PSC-PhRPs may restore their functionality by neuroprotection or another unknown mechanism. To account for this possibility, behavioral tests were conducted following dark adaptation and under dim light; designed to target transplanted rod photoreceptor sensitivity rather than residual cones in host animals. Additional support for the restoration of phototransduction by donor cells comes from the observation that markers expressed by transplanted cells are consistent with rod light-sensitivity and donor-host connectivity. Furthermore, the consistent number of residual cone photoreceptors in mice of the sham and treatment groups, indicates that functional improvement observed in the treatment groups was not caused by the rescue of host cones from further degeneration. While unlikely due to the age of mice, the possible involvement of a therapeutic effect exuded by transplanted human cells on residual cone function should not be ruled out, and may be further studied as a novel therapeutic avenue of cell replacement.

We found an improvement in optomotor response in treated animals which was of a similar magnitude using either ESC or iPSC derived cells, corroborating *in vitro* observations of similar differentiation efficacy in these cell lines. Both the OMR response and light avoidance behavior correlated with the number of surviving donor cells, which is more in keeping with a direct response from these cells as opposed to random events. A variation in the number of surviving cells could represent variation in transplantation and/or delivery of immunosuppression to different animals; however, it is possible that the subretinal space was a more permissive environment for cell survival in animals which shared a preexisting inherent trait such as slower cone degeneration. Survival of non-functional cones in end-stage RP patients has been previously documented^55^. Hence further exploration of this question would be important in identifying ideal time points for photoreceptor transplantation in humans and synchronized retinal differentiation will correspondingly allow exploration of the most suitable developmental stage for integration and further improve transplantation efficiency.

Taken together, the data presented here provide an important step towards manufacturing high quality transplantable human photoreceptors from human PSCs, as well as novel evidence that these cells may possess the potential for photoreceptor replacement therapy in blind patients.

## Materials and Methods

### Retinal photoreceptor cell differentiation

To induce neural differentiation, 90% confluent human pluripotent stem cells were split onto the Matrigel at the 1:25 ratio. When cell colonies grew to about 50–100 cells, cell differentiation were initiated by directly switching cell culture medium from mTESR1 to retinal induction medium (RIM) containing DMEM/F12, N2 and B27 serum-free supplements, 100 units/ml penicillin, 100 μg/ml streptomycin (Life technology), 0.45% glucose (Sigma), 20 μg/ml human insulin (Roche) and 50 ng/ml human Noggin (Peprotech) at 37 °C/5% CO_2_. On day 5, RIM medium was switched to neural differentiation medium plus (NDM+), containing Neurobasal, N2 and B27 serum-free supplements, 100 units/ml penicillin, 100 μg/ml streptomycin, Glutamax, MEM Non-essential amino acid (Life technology), 0.45% glucose and 50 ng/ml human Noggin for two weeks. On Day 19, cells were mechanically lifted using a scraper (Corning) and plated into ultra-low attachment dishes in NDM minus (NDM^-^, without Noggin). Cells formed neural spheres in the suspension culture. After four days (≈day 23), neural spheres were collected and plated on Matrigel coated surface in NDM-. Cells were maintained in NDM- until the desired maturation stage for a given experiment.

To generate mature photoreceptor-like cells, photoreceptor progenitors (PhRP) were dissociated into single cells using accutase digestion and plated on Matrigel coated dishes at the density of 10^5^/cm^2^. Cells were cultured in photoreceptor differentiation medium containing neurobasal, N2 and B27 serum-free supplements, 100 units/ml penicillin, 100 μg/ml streptomycin, Glutamax, MEM none essential amino acid (Life technology), 0.45% glucose, 10 ng/ml human BDNF, 10 ng/ml human CNTF, 2 μM retinoid acid and 10 μM DAPT for two weeks.

### Animals

Wild type (WT) C57BL/6 mice were provided by the Biomedical Sciences division, University of Oxford and C3H/HeNHsd-Pde6b^*rd1*^ (*rd1*) mice were purchased from Harlan Laboratories (Hillcrest, UK). Mice were all female and were 10–12 weeks old at the time of intraocular injection. All animals were housed under standard 12:12 hour light/dark cycle, with food and water available ad libitum. Procedures were performed according to the UK Home Office guidelines on the Animal (Scientific Procedures) Act of 1986 and were approved by the University of Oxford Animal Ethics Committee and in accordance with the Association for Research in Vision and Ophthalmology statements on the care and use of animals in ophthalmic research.

### Cell counts

The number of surviving cells per eye was determined by counting GFP positive cells in serial 18 μm non-overlapping sections through each eye. Cells were considered for analysis if they resided in the subretinal space or within the outer nuclear layer. Cells residing in the vitreous were not included in quantification.

### Optomotor response (OMR)

Mice were placed on a raised platform in the center of a custom built rotating cylinder with a square-wave grating of black vertical stripes corresponding to 0.1 cycles per degree (cpd) spatial frequency. Mice were dark adapted for >12 hours prior to the procedure and testing was conducted in a dark room, with the cylinder illuminated from above by a custom LED array emitting a dim 510 nm green light (150 nW · cm^−2^ · s^−1^, approximately 10 lux at the platform). Thorough cleaning with 70% ethanol was conducted before each test and the tester was masked to the treatment. Mice were first habituated to the environment for 1 min during which the drum remained stationary and mice were free to explore the platform. Each experimental run consisted of 1 minute clockwise and 1 minute anti-clockwise rotation, divided into alternating 30 second periods^52^. The experimental run was repeated three times for each animal. The rotation of the square-wave grating elicited an optomotor response, with a single response consisting of a slow head-tracking motion in the direction of the drum’s rotation followed by a rapid repositioning of the head to a central position. Mean wild-type (WT) response was derived by testing age matched mice (n = 8). Behavior was recorded by use of a digital camera mounted directly above the central platform. All experiments were conducted by a female researcher and the number of head tracks was quantified manually by two independent scorers, blinded with regards to treatment and averaged between the three experimental runs. To eliminate any bias by observers, the observers were blind to the fact that a single eye was injected rather than both eyes and watched videos of coded WT animals, sham animals and treated animals in a randomized mixed order.

### Light avoidance response

Mice were tested in a partitioned arena with equally sized dark and light chambers connected by an aperture through which the animal were able to transition freely, as previously described^4^. Briefly, mice were dark adapted >12 h before testing and testing was conducted in a dark room. The light chamber was lit by a LED array suspended above the chamber emitting dim green light centered at 510 nm (maximal illumination of 150 nW · cm^−2^ · s^−1^, approximately 10 lux at the arena floor). Both chambers were thoroughly cleaned with 70% ethanol before each test and the tester was masked to treatment group. Pupils were dilated 10 min prior to testing with one drop of 1% atropine. Mice were placed in the middle of the lit chamber facing away from the connecting aperture. Each trial lasted 10 min, and all mice were test-naive (a single trial per mouse). Entrance to a chamber was recorded only when all four paws had crossed into that chamber. Light avoidance was measured by the percentage of time spent in the dark chamber. The number of full body transitions between chambers and distance travelled within the lit chamber were recorded as a measure of anxiety-related behavior. Mean wild-type (WT) response was derived by testing age matched mice (n = 8). Data were recorded by a digital camera mounted above the lit chamber and calculated by ANY-Maze video tracking software (Version 4.5 http://www.anymaze.com/).

### Statistical Analyses

All measures are presented as mean and standard error of the mean (SEM).

### Supplemental experimental procedures

Culture of undifferentiated hPSCs, gene expression analyses, flow cytometry analysis, AAV vector generation, cell cryopreservation, cell recovery and transfection, cell transplantation, Scanning Laser Ophthalmoscopy, tissue collection and processing, immunocytochemistry of *in vitro* cells, retinal immunohistochemistry, light microscopy, confocal microscopy, immune suppression and detailed statistical analysis are reported in the Supplemental Experimental Procedures.

## Additional Information

**How to cite this article**: Barnea-Cramer, A. O. *et al*. Function of human pluripotent stem cell-derived photoreceptor progenitors in blind mice. *Sci. Rep.* **6**, 29784; doi: 10.1038/srep29784 (2016).

## Supplementary Material

Supplementary Information
