# Supplementary material for: Function of human pluripotent stem cell-derived photoreceptor progenitors in blind mice
Source: Sci Rep. 2016 Jul 13;6:29784. doi: 10.1038/srep29784 (PMC4942817; doi:10.1038/srep29784)
Supplement: Supplementary Information [file srep29784-s1.pdf]

Supplementary Information

Function of human pluripotent stem cell-derived photoreceptor progenitors in blind mice

Alona O. Barnea-Cramer[1*], Wei Wang[2*], Shi-Jiang Lu[2*], Mandeep S. Singh [1,3], Chenmei Luo[2], Hongguang Huo[2], Michelle E. McClements[1], Alun R. Barnard[1], Robert E. MacLaren[1,3,4#] and Robert Lanza[2,#],

* Joint first authors
[#] Joint corresponding authors

1. Nuffield Laboratory of Ophthalmology, University of Oxford
2. Astellas Institute for Regenerative Medicine, 33 Locke Dr, Marlborough, MA 01752
3. Moorfields Eye Hospital NHS Foundation Trust NIHR Biomedical Research Centre
4. Oxford University Hospitals NHS Trust Biomedical Research Centre

Supplementary Tables and Figures

Table S1. Evaluation of eye field progenitor differentiation from multiple human ESC and iPSC lines

Cell lines	Percentage of PAX6/RX1positive cells
ESC lines (N=3), blastocyst: H1, H7 and H9.	92%-98%
ESC lines, single blastomere technology (N=3): MA01, MA09 and NED7.	94%-99%
iPS lines, Episomal Vector (N=4): iPS-1, iPS-2, iPS-3 and iPS-4	90%-99.6%
iPS lines, mRNA (N=2): HA and BJ	92%-98%

Table S2. Number of surviving cells, optomotor response (OMR) and behavioral light avoidance per animal

Animal	Treatment	Surviving cells	OMR (Head tracks)	% Light avoidance
1	H9-ESC	11302	1	11.80
2	H9-ESC	4296	0	25.62
3	H9-ESC	7050	3	19.45
4	H9-ESC	20230	5	71.18
5	H9-ESC	7240	2	33.18
6	H9-ESC	16549	2	59.15
7	H9-ESC	19281	5	65.57
8	H9-ESC	17431	5	50.52
9	HA-iPSC	3289	2	30.47
10	HA-iPSC	5649	2	22.28
11	HA-iPSC	10947	4	70.15
12	HA-iPSC	9835	4	51.70
13	HA-iPSC	11542	2	57.80
14	HA-iPSC	14002	5	59.77
15	HA-iPSC	19018	7	57.82
16	HA-iPSC	16992	3	72.85

Supplemental Figure legends

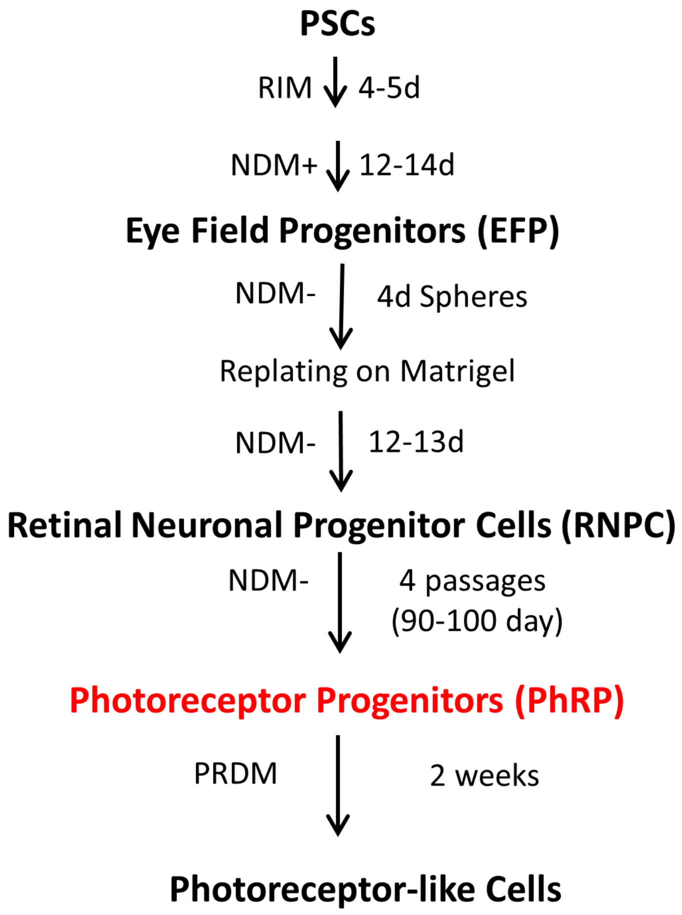

Figure S1. Schematic of the procedure for the generation of RNPCs and PhRPs from human PSCs.

PhRPs used in *rd1* study were highlighted in red. RIM, retinal induction medium; NDM, neural differentiation medium with (+) or without (-) Noggin supplement; PRDM, photoreceptor differentiation medium.

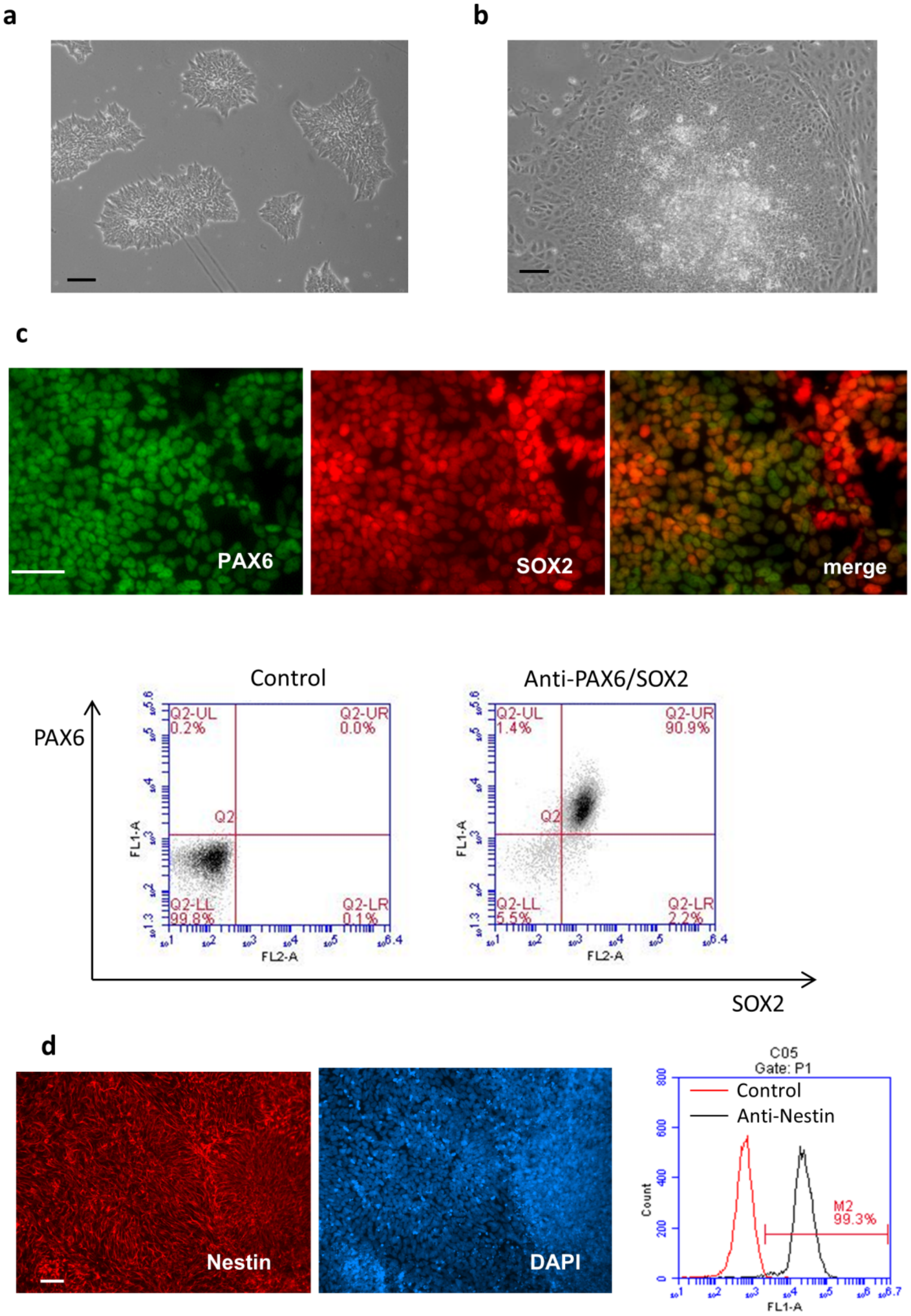
a
b
c
PAX6
SOX2
merge
Control
Anti-PAX6/SOX2
PAX6
SOX2
Q2-UL 0.2%
Q2-UR 0.0%
Q2-LL 99.8%
Q2-LR 0.1%
Q2-UL 1.4%
Q2-UR 90.9%
Q2-LL 5.5%
Q2-LR 2.2%
Q2
FL1-A
FL2-A
d
Nestin
DAPI
C05
Gate: P1
Control
Anti-Nestin
M2
99.3%
Count
FL1-A

Figure S2. Morphological changes of human ESC culture during in vitro neural induction.

a, After 1 day after cultured in retinal induction medium, cells at the colony margin were column-shaped. **b,** An example of a differentiating ESC colony after 7 days of in vitro differentiation. **c**, Top panel, immunofluorescence co-staining of PAX6 and SOX2 on cells after 10 days of in vitro differentiation. bottom panel, quantification of PAX6 and SOX2 double positive cells by flow cytometry analysis, which shows > 90% of them expressing both PAX6 and SOX2 proteins**. d,** Immunofluorescence and flow cytometry quantification of nestin expression after 10 days of in vitro differentiation. Scale bar, 50 μm.

a

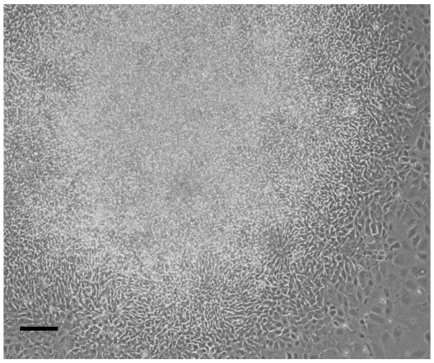

b

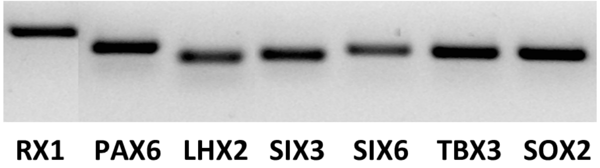

c

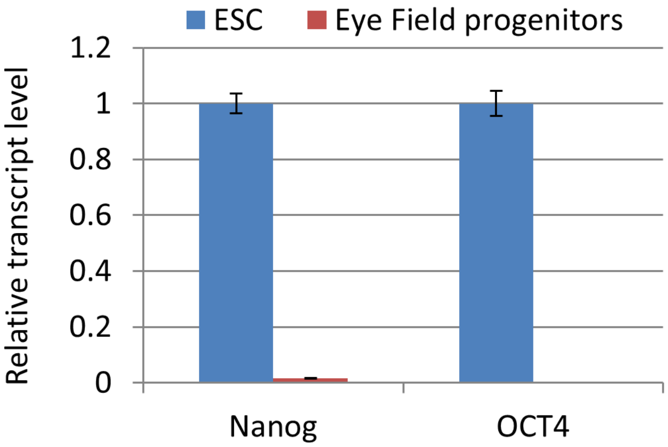

Figure S3. Morphological characteristics of hESC-derived eye field progenitors.

a, An example of a differentiating eye field progenitor colony after 13 days of in vitro differentiation. **b**, RT-PCR analysis of RX1, PAX6, LHX2, SIX3, SIX6, TBX3 and SOX2 on cells after 13 days of differentiation. **c**, Quantitative RT-PCR analyses of ESC pluripotent markers on H9 human ESCs and on day 13 eye field progenitors. Scale bar, 50 μm.

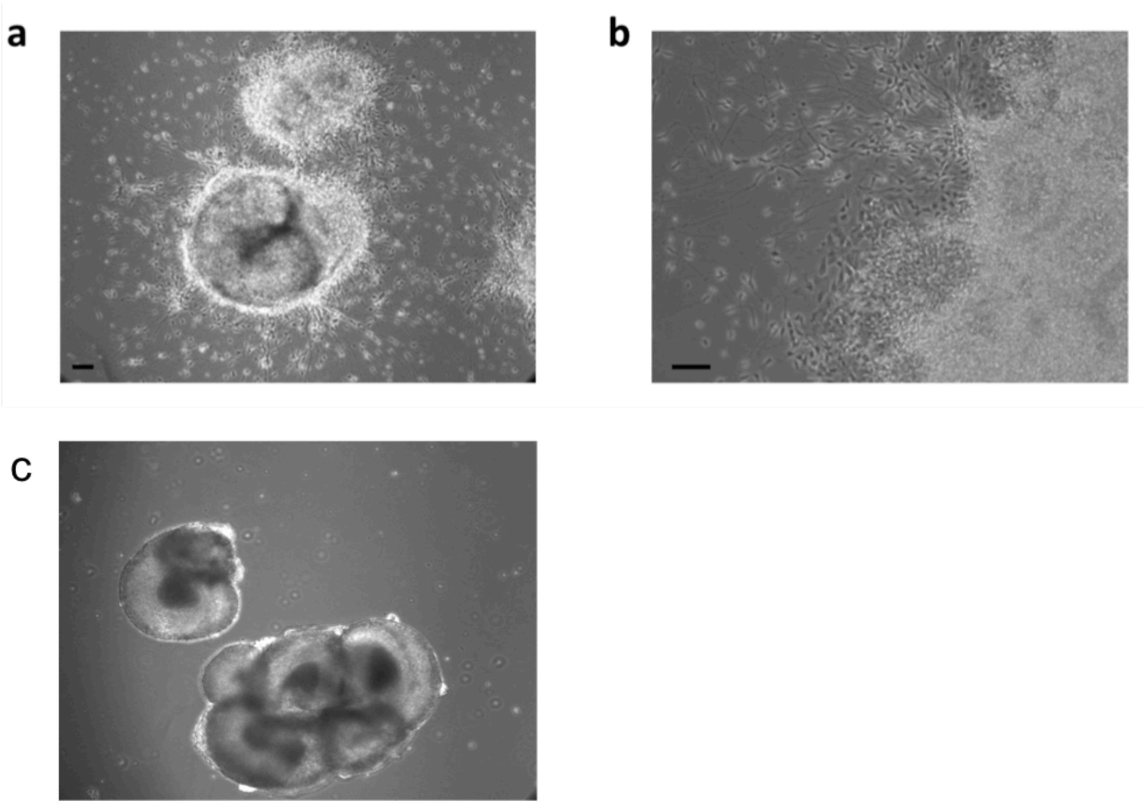

Figure S4. Morphological characteristics of hESC-derived retinal neural progenitor cells.

a, Spheres on Matrigel surface 48 hours after plating eye field progenitors. Note: neurons were migrating out from cell aggregates. **b**, Neural rosettes were formed after 7 days in culture on Matrigel surface. **c**, RNP spheres formed eyecup like structures in suspension cultures. Scale bar, 50 µm.

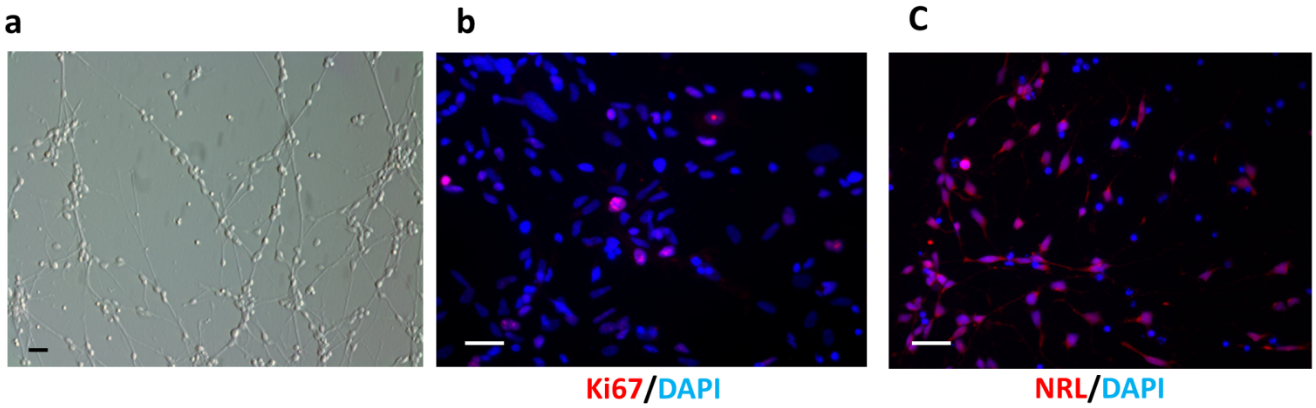

Figure S5. Generation of photoreceptor progenitors from human PSCs. a, An example of photoreceptor progenitors at day 110 after initiation of in vitro differentiation. Scale bar, 50 µm. **b** Immunofluorescence staining of a cell proliferation marker Ki67, in hESC-derived PhRPs at day 100 after in vitro cell differentiation. **c**, immunofluorescence staining showing the expression of NRL in photoreceptor progenitors derived from human iPS cells; Scale bar, 20 µm.

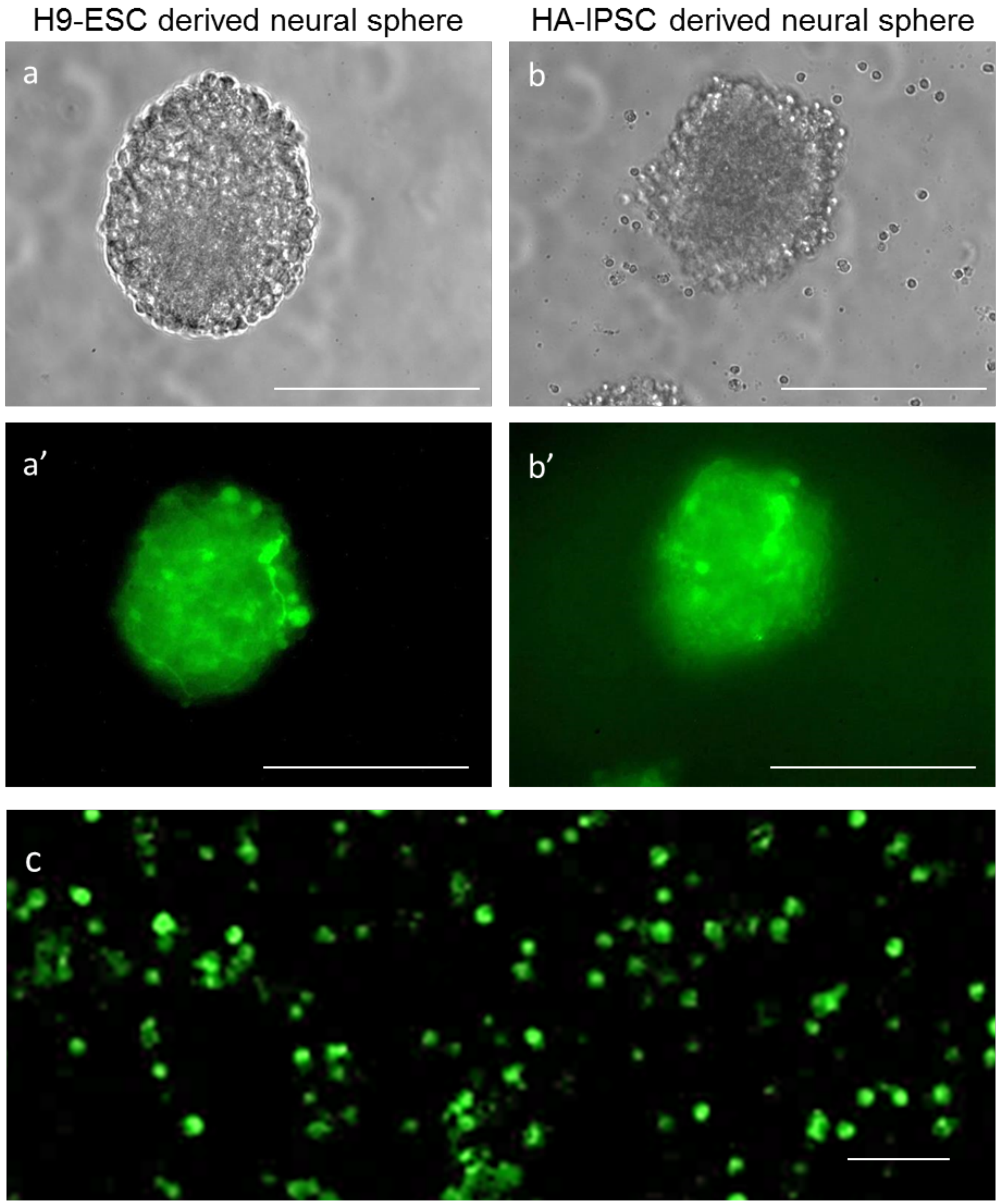

Figure S6. Specific transduction of human donor cells in WT and rd1 mice

a. H9-ESC-PhRP sphere and **b**, HA-iPSC-PhRP spheres in vitro. GFP was induced by rAAV2 Y444F driving GFP by the photoreceptor-specific rhodopsin kinase promoter. GFP expression was achieved in vitro 7 days post transduction of PhRPs derived from both H9-ESC **a'**. and HA-iPSC **b'**. Dissociated human PhRPs (shown in **c**) were transplanted in WT mice 48 hours post transduction. Scale bar, 20µm.

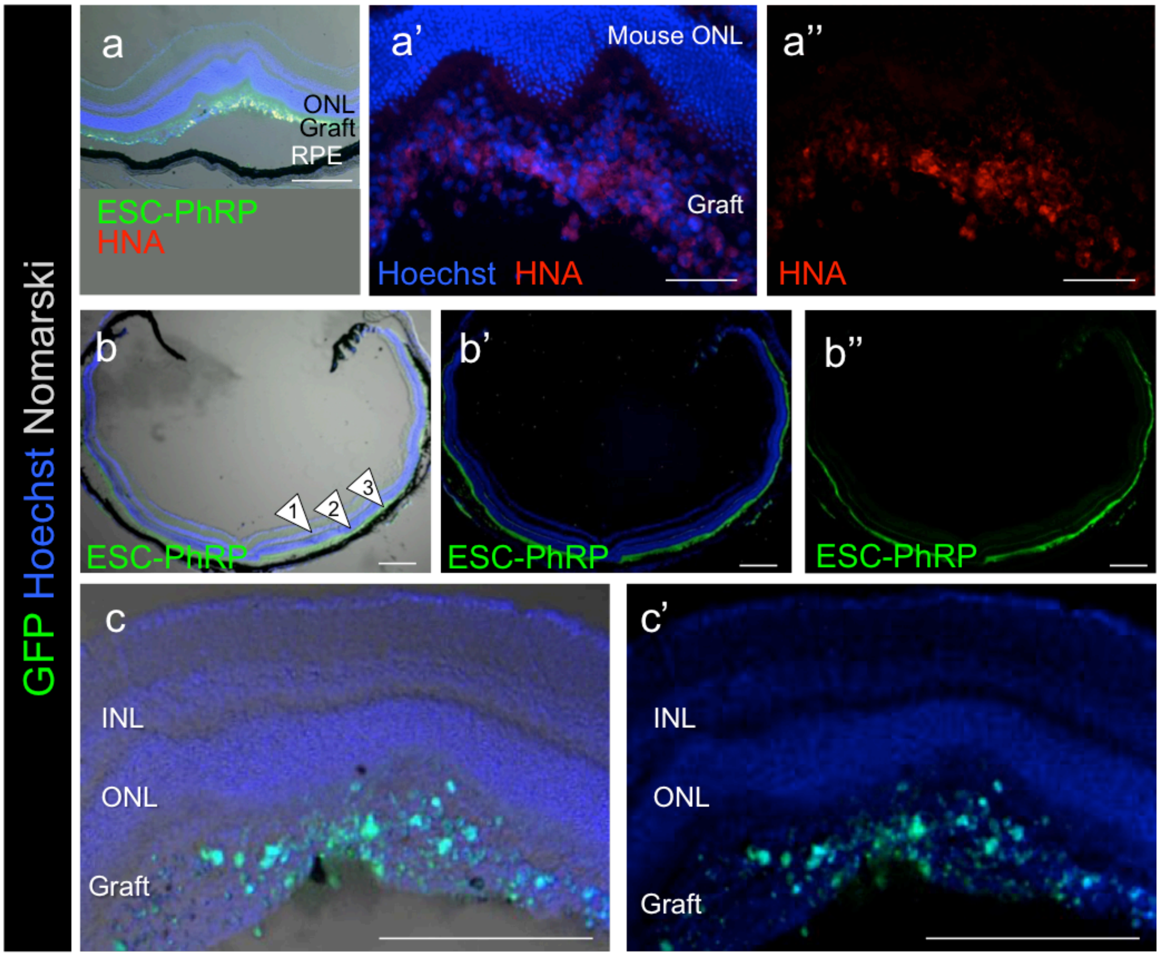

Figure S7. In vivo GFP expression in transplanted human cells 7 days post transplantation.

a. Human ESC-PhRPs survive in the subretinal space of WT mice and express both AAV-driven GFP and human nuclear antigen (HNA); **a'-a''**. Magnified image of the mouse ONL and human graft stained with HNA, confirming the identity of human derived cells. **b-b''**, Low-power image of an entire retinal section, showing GFP-positive ESC-PhRPs in the subretinal space and no GFP transduction of the host INL (white arrow 1), ONL (arrow 2) or RPE (arrow 3). c-c', Magnified extract from image b, showing the layers of the host retina and the GFP-positive ESC-PhRP graft in the subretinal space. Note that GFP is confined to the grafted cells without transduction of the host ONL or INL. Scale bar, 100µm. INL, inner nuclear layer; ONL, outer nuclear layer; RPE, retinal pigment epithelium; HNA, human nuclear antigen.

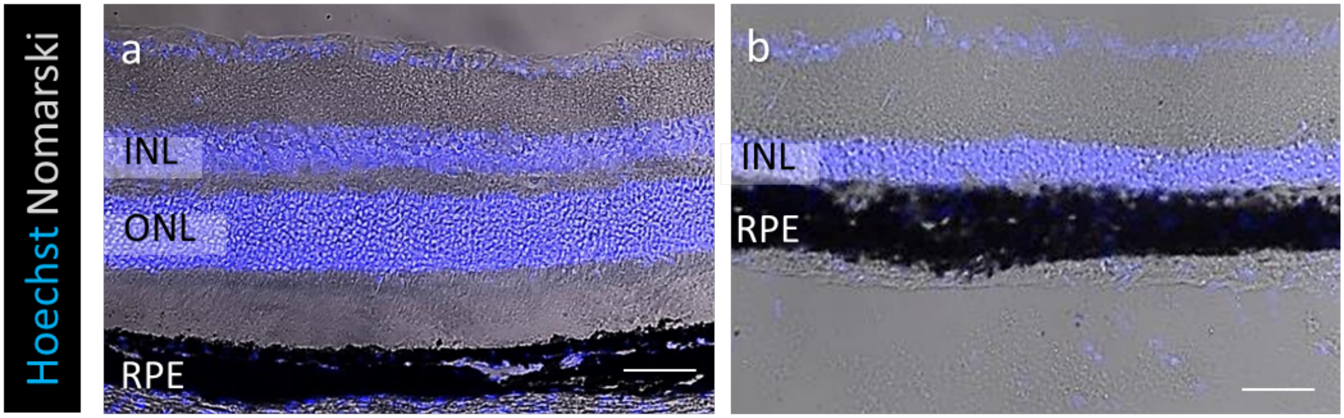

Figure S8. Morphological comparison of WT and rd1 retinae at 10 weeks of age

a, The retina of a wild type mouse with an intact INL and ONL; **b**, The retina of an rd1 mouse shows the absent ONL which is the layer of cells to be replaced by human PhRPs. Scale bar, 100 µm. INL, inner nuclear layer; ONL, outer nuclear layer; RPE, retinal pigment epithelium.

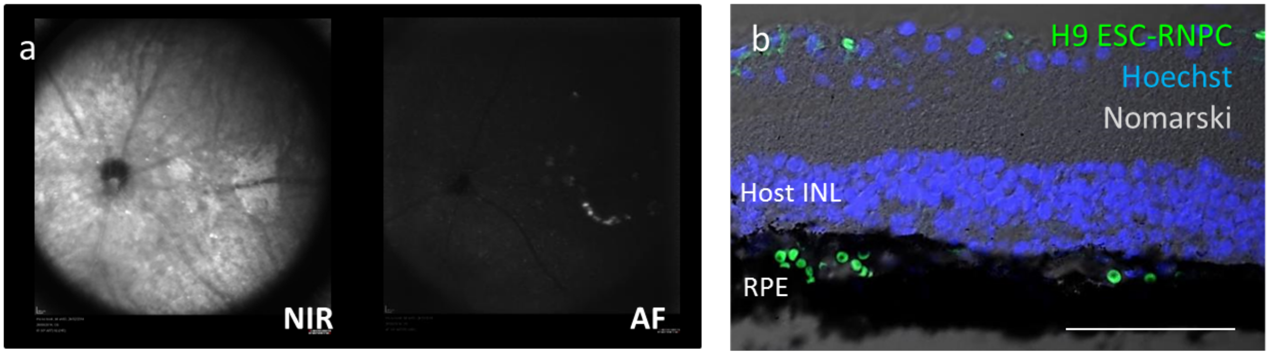

Figure S9. Retinal neuronal progenitor cells (RNPCs) do not survive in the rd1 subretinal space and host retinal transduction is not observed following transplantation of rAAV2.GFP infected cells

In order to control for cell survival and in vivo transduction of the host retinae by free AAV particles, rd1 mice received transplantation of retinal neuronal progenitor cells (RNPCs) transduced with rAAV2 expressing GFP under a ubiquitious CMV promoter. These cells did not survive in the subretinal space of rd1 mice, and a small number of GFP-positive cells was observed in only 2 of 5 treated animals by scanning laser ophthalmoscopy (SLO) (**a**) or by histology (**b**). GFP observed within the RPE does not represent cell bodies (as indicated by the absence of Hoechst nuclear staining) and are most likely result of auto-fluorescent tissue. The absence of GFP-positive cells in eyes of this control group indicates that GFP observed in vivo in PhRP treated animals was indeed a marker for transplanted human PhRP cells. Scale bar, 100 μm. NIR, near-infrared; AF, Autofluorescence; INL, inner nuclear layer; RPE, retinal pigment epithelium.

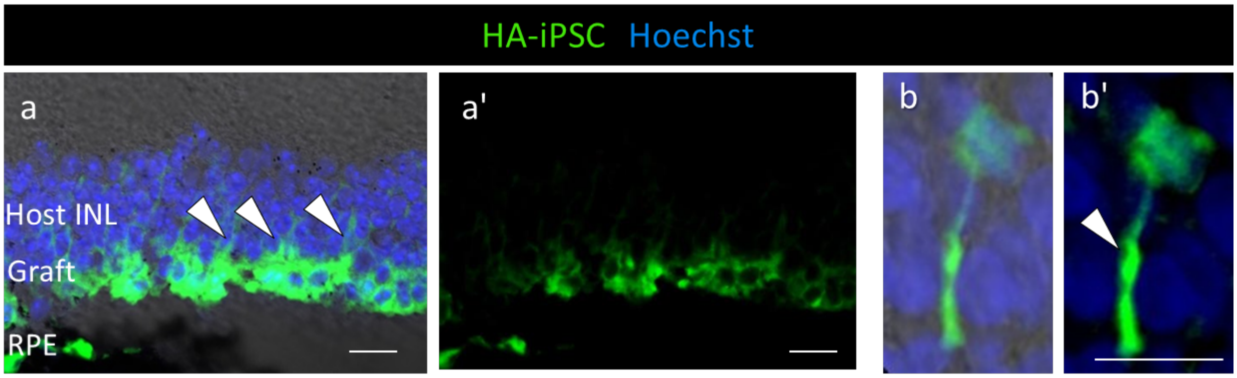

Fig S10. Morphology of grafted human cells

Transplanted cells interact with host by extending terminals (white arrowheads in a) into the host INL (**a-a'**) and developing processes (white arrowhead in b'), as observed in maturing photoreceptor cells in retinal flatmounts (**b-b'**). Scale bar, 20μm. INL, inner nuclear layer; RPE, retinal pigment epithelial.

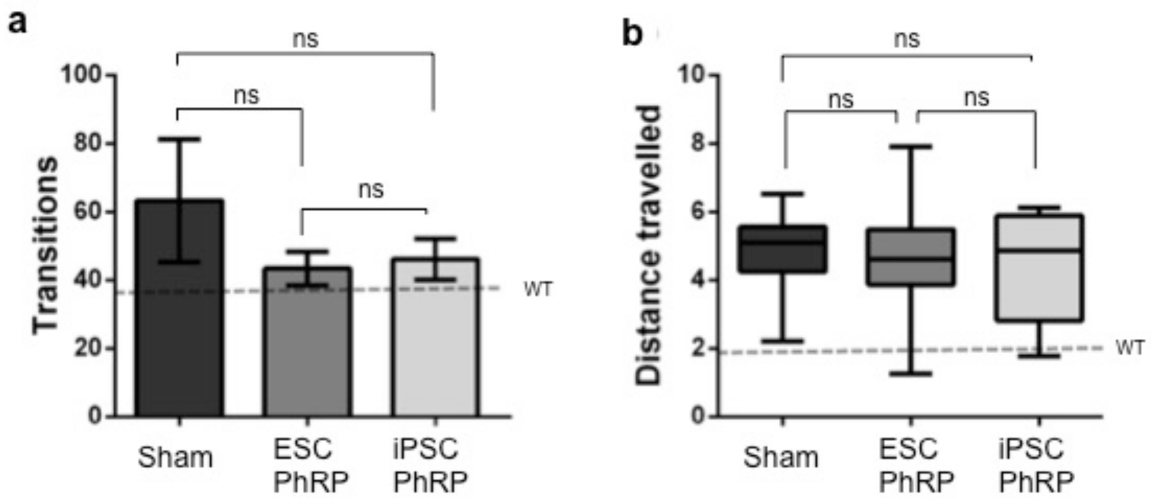

Figure S11. Control measures for functional improvement of vision

In order to control for differences in behavior of the three treatment group in the light avoidance assay, transitions between the arena chambers and the distance travelled by animals within the lit chamber were also assessed during the experiments as behavioral measures of anxiety in *rd*1 mice. There were no differences between the three groups in (**a**) mean number of transitions between the light and dark chambers (F=0.9014, p=0.4211 [ns]) or (**b**) the mean distance (meters) traveled within the lit chamber (F=0.1297, p=0.8790, ns) throughout the test. Dashed line represents the mean response of age-matched wild-type mice. One way ANOVA, n=8 per group, ns, non-significant.

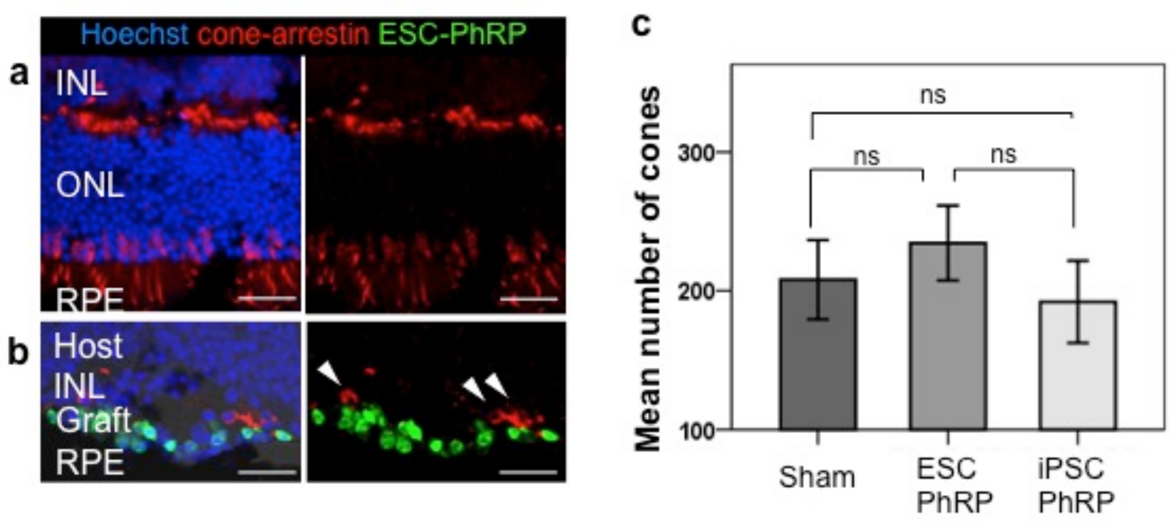

Figure S12. Control measures for morphology and number of residual host cones

To account for the possibility of improvement in visual function resulting from host cone neuroprotection by transplanted cells, the number of residual cones was assessed by cone arrestin staining. Image **a**. shows cone arrestin staining of an adult WT retina, with cone bodies, processes and inner and outer segment stained (red). **b** Residual cones (red) in the adult *rd1* mouse three weeks after transplantation of ESC-PhRP (green). Host cone cells show abnormal morphology and absence of inner and outer segments. **c**. Residual host cones were quantified three weeks following transplantation, and no difference was found in the number of residual host cones between mice treated with ESC-PhRP, iPSC-PhRP or sham injection (F=0.56, p=0.58, ns; One way ANOVA, n=5 specimens per group). Error bars represent ±1 S.E.M. INL, inner nuclear layer; ONL, outer nuclear layer; RPE, retinal pigment epithelial; ns, non-significant.

Supplementary Methods

Culture of undifferentiated human PSCs

Human ESC lines (H1 (WA-01), H7 (WA07) and H9 (WA-09), derived from blastocyst; MA01, MA09 and NED7, derived from single blastomeres (passages 35–45) and iPS cell lines (HA and BJ, derived from human dermal fibroblasts using non-integrating 6 factor mRNA reprogramming kit [1]; iPS-1, iPS-2, iPS-3 and iPS-4, derived from human fibroblasts using episomal vectors; passages 26–32) were maintained on Matrigel® (BD Biosciences, CA) in mTESR1 medium (Stemcell Technology, Vancouver, BC, Canada).

Gene expression analyses

Total RNA was extracted from cells using an RNeasy kit (Qiagen) following the instruction of user's manual. First-strand cDNA was synthesized with random hexamers using the SuperScript III reverse transcription (RT)-PCR system (Life technology). Reverse transcriptase polymerase chain reaction (RT-PCR) for eye field transcription factors were performed using the following primers: PAX6, F-TCTAATCGAAGGGCCAAATG R-TGTGAGGGCTGTGTCTGTTC; RX1, F-CGGCCAACAAGAAGATGAGT R-GCCATGGAGTTCAAGTCGTT; LHX2, F-TAGCATCTACTGCAAGGAAGAC R-GTGATAAACCAAGTCCCGAG; SIX3, F-GGAATGTGATGTATGATAGCC R-TGATTTCGGTTTGTTCTGG; TBX3, F-TCCGACTCCGTCTCTCTCTC R-CAAATCTTGCTGGGCTCTTC; SIX6, F-TCCCATGGGTATTTCACGTC R-CACACAGAACCCATCACCAC; and SOX2, F-GGAGCTTTGCAGGAAGTTTG R-AATTCAGCAAGAAGCCTCTCC. For real-time RT-PCR, transcripts were assayed using commercially available TaqMan gene expression assays (Life technology) for mash1 (Hs04187546_g1), RORβ (Hs00199445_m1), rhodopsin (Hs00892431_m1), cone-opsin (Hs00241039_m1) and recoverin

(Hs00610056_m1), with the data normalized to GAPDH (4352934E). Each data point represents nine technical replicates from three independent biological samples.

Flow cytometry analysis

The expression of eye field and retinal neuronal and photoreceptor cell markers were quantified by Flow cytometry on an Accuri C6 flow cytometer (BD) according to standard procedures. Briefly, single cells were harvested by trypsin digestion. Cells were fixed and permeabilized with intracellular fixation and permeabilization buffer set following the instruction of the kit manual (e-bioscience). Primary antibodies were freshly prepared in PBS buffer with 5% FBS. Typically 5×10^5 cells were used for antibody labeling. Cells were stained in a 100 µl primary antibody cocktail for 1 h on ice, then washed twice with buffer, and incubated in 100 µl buffer supplemented with appropriate Alexa Fluor® 488 or Alexa Fluor® 647-conjugated secondary antibodies for 30 min on ice. Isotype mouse immunoglobulin G (IgG) or no primary antibody served as a negative control. Data was analyzed using BD Accuri C6 software.

AAV vector generation

The Y444F mutation was created in the Rep2Cap2 plasmid using the QuikChange II site-directed mutagenesis kit (Agilent Technologies) as per instructions with the HPLC purified forward primer: CAGTACCTGTATTTCTTGAGCAGAACAAACAC and reverse primer: TTTGTTCTGCTCAAGAAATACAGGTACTGGT. The transgene plasmid (pTransgene) was generated by isolating the 199bp human rhodopsin kinase promoter (GRK1) fragment previously described [2]. This was attached to the exon/intron elements of the CAG promoter using the SPLICE technique [3]. In order to do this, the GRK1 reverse primer contained the first few bases of the exon fragment and similarly, the forward primer of the exon fragment contained the last few bases of the GRK1 sequence. Restriction sites are underlined.

hGRK1 forward primer: TCGGTACCGGGCCCCAG; GRK1 reverse primer: GCGCGCAGCGACTCCCCCGGGGCTGACA; Exon forward primer: TGTCAGCCCCGGGGGAGTCGCTGCGCGC Intron reverse primer: GTGCTCAGCAACTCGGGGAG. The resulting GRK1/exon-intron amplicon was 710bp. The plasmid pUF6.1 containing CAG.GFP.SV40pA.bGHpA between AAV2 ITRs was disrupted by KpnI and BlpI digest, resulting in the removal of CMV enhancer, CBA promoter, exon element and some intron element from the CAG. The amplified GRK1pr/exon-intron fragment was then inserted into the digested plasmid via the KpnI and BlpI sites. The insertion resulted in the replacement of CMV enhancer and CBA promoter with GRK1 promoter but no loss of CAG exon/intron sequence. The resulting transgene was 2.7kb long containing GRK1pr.exon.intron.GFP.pA. ITR integrity was checked by partial sequencing and XmaI digestion. PEI triple transfection was conducted on 293T cells with pHelper, pRep2Cap2Y444F and pTransgene. Cell lysates were collected three days post-transfection and subsequent AAV purification performed using iodixanol gradients [4]. Isolated AAV were concentrated using Amicon Ultra-15 100K centrifugal units (MerckMillipore) and assessed for purity by SDS-PAGE.

Cell cryopreservation

For long term storage and cell shipment, PhRP spheres were cryopreserved in an-animal-free cell cryopreservation buffer, Cryostor CS10 (BioLife Solutions, Inc). 5 million cells were frozen in a final volume of 1 ml of Cryostor CS10 medium. Then stored at -80C for 2 days, and transferred to liquid nitrogen. Frozen cells were rapidly thawed in a 37°C water bath and cells viability was assayed by Trypan Blue (sigma) exclusion.

We routinely check cell viability after cell cryopreservation. Viability was tested immediately post thawing, again at 48 hours and finally prior to transplantation. Using the described method, we typically obtained 70-85% viable cells post thawing. Only batches with over 75% viable cells were used for further application. Following media replacement and removal of dead cells, we tested

viability again at 48 hours and immediately prior to transplantation. Over 90% cell viability was measured in cohorts at 48 hours and at the day of transplantation.

Cell recovery and transduction

Frozen human ESC and iPSC-derived PhRPs were maintained in vapor phase liquid nitrogen storage. 72 hours prior to transplantation cells were thawed, transferred to 10 cm Ultralow binding plates (Corning®) and maintained in NDM- at 37 °C, 5% CO_2 and 90% humidity to form PhRP spheres. After 24 hours in culture, cells were transduced via a capsid-mutant recombinant serotype 2 adeno-associated virus (rAAV2 Y444F), expressing GFP under the photoreceptor-specific human rhodopsin kinase promoter. The cells were transduced as neural spheres, rather than single cells as the viability of spheres is greater than single cells post-thaw. Virus was added to cells in culture at a multiplicity of infection (MOI) of approximately 10^5 viral genomes (vg)/cell. The media were changed after 48 hours and cells were imaged daily to detect GFP expression. In order to prevent potential cell loss or mutation during prolonged culture we aimed to reduce the culture period for human PhRP rather than waiting for GFP to be expressed in vitro prior to transplantation. Hence, for transplantation, 48 hours after transduction (before peak GFP expression) PhRP spheres were dissociated with Accutase (Innovative Cell Technologies, Inc.) and filtered through a 40μm cell strainer (BD FalconTM). Cells were rinsed in 5 ml PBS, centrifuged in sterile tube at 70 RCF (x2), to eliminate free AAV particles from media prior to transplantation and were then re-suspended to a final concentration of 10^5 cells/μl balanced salt buffer for injection.

Calculation of transduction ratio

Dissociated cells were imaged every 24 hours by light microscope and counted using ImageJ software (Version 1.47, National Institute of Health, http://rsb.info.nih.gov/ij/index.html). Average of cells per field of view was calculated as the mean number of cells counted per view on a 20x microscope objective, with 5 fields of view assessed per plate to determine a mean cell

count. Counting was performed manually and cells were counted in vitro under a light microscope. The total number of cells in the plate, as well as the amount of GFP+ cells was quantified and averaged between 5 fields. The transduction ration was calculated as the percent of GFP+cells /all cells per field.

Viability assessment

Cell viability was also determined by use of Trypan blue (0.4%, Life technologies) by mixing 20 µl trypan blue with 20 µl 1:100 cell suspension. In viable cells, the trypan blue dye does not pass through the cell membrane and is not absorbed, however it traverses the membrane of dead cells, and these are shown labelled with a blue dye under the microscope. The percent of viable cells was quantified using a haemocytometer and cell suspensions were used when established as over 90% viable 48 hours after being thawed as well as immediately before transplantation.

Anesthesia

For intraocular injections and in vivo imaging procedures general anesthesia was induced by a single intraperitoneal injection of Vetalar (Ketamine Hydrochloride, 80 mg/kg) and Rompun (xylazine, 10 mg/kg) and pupils were fully dilated with 1% tropicamide and 2.5% phenylephrine hydrochloride eye drops (both Bausch & Lomb, Kingston-Upon-Thames, UK). Anesthesia was reversed following transplantation by intraperitoneal injection of antisedan (Atipamezole, 2 mg/kg body weight). Animals in the treatment and sham transplantation groups were housed together to reduce variability between treatment groups.

Cell transplantation

Transplantations were performed by subretinal injection under direct visualization using an operating microscope (M620 F20, Leica, Wetzlar, Germany). The pupils of 10-12 week old mice were dilated as described above and a liquid gel (Viscotears, Novartis, Frimley, UK) was applied to the eye. A 6mm circular cover glass was positioned over the cornea to allow visualization of

the retina. Cell suspensions were transplanted subretinally using a Hamilton syringe and a 10mm 34-gauge needle (65N, Hamilton AG) inserted into the subretinal space though the sclera as previously described [5]. 2 µl of diluted cells (Approximately 2×10^5 cells were transplanted in each injection) or buffer (PBS) was delivered unilaterally into the subretinal space of the right eye.

Scanning Laser Ophthalmoscopy

Autofluorescence (AF) imaging was performed 3 weeks post transplantation using a confocal scanning laser ophthalmoscope (cSLO; Spectralis HRA, Heidelberg Engineering, Heidelberg, Germany) as previously described[6]. Animals were anaesthetised and pupils were dilated as above. A contact lens was placed on the cornea using a viscous gel (0.3% w/v hypromellose, Matindale Pharmaceuticals, Romford, UK) to improve image quality, and the mouse was place on an imaging platform. The near-infrared (NIR) mode (820 nm laser) was used to achieve camera alignment at the confocal plane of the neural retina. GFP expressing cells were imaged using the autofluorescence (AF) mode (480 nm) using a 55° lens with a standardized detector sensitivity of 70 and automated real-time averaging.

Tissue collection and processing

Eyes were fixed in 4% paraformaldehyde (PFA, Thermo Fisher, Loughborough, UK) in PBS and the cornea and lens were removed before overnight incubation in 4% PFA. Fixed eyes were cryoprotected in a 10-30% sucrose gradient, then washed in PBS and embedded in optimal cutting temperature (OCT) compound (Tissue-Tek, Sakura Finetek, The Netherlands) and frozen in liquid nitrogen. Cryosections (18 µm) were cut and affixed to poly-L-lysine coated glass slides (Polysine®; Thermo Scientific, Loughborough, UK) for immunohistochemistry and further analysis.

In vitro immunocytochemistry

Cells were fixed in 4% paraformaldehyde for 30 min at room temperature. Samples were blocked with 5% normal goat or donkey serum (Jackson ImmunoResearch) and 0.3% Triton X-100 in PBS at room temperature for 1 h, followed by incubation with primary antibodies at room temperature for 1 h. There following antibodies and sources were used: PAX6-specific antibody (1:600, DSHB); SOX2-specific antibody (1:400, Cell Signaling, 3579); RX1/RAX-specific antibody (rabbit polyclonal, 1:100, Abcam, ab23340); human nestin-specific antibody (1:500, R&D, mab1259), TRβ2-specific antibody (1:200, abcam, ab53170); NRL-specific antibody (1:200, Sigma, SAB1100608); Mash1-specific antibody (1:100, Abnova, H00000429-M02); RORβ-specific-Rabbit antibody (1:50, Millipore, Cat# AB9482); RXRϒ-specific antibody (1:100, abcam, ab15518); CRX-specific antibody (1:25, Santa Cruz, SC-30150); NR2E3-specific antibody (1:50, R&D, PP-H7223-00), opsin-specific (red/green) antibody (rabbit polyclonal, 1:300, Millipore, AB5405); monoclonal Rhodopsin-specific (1:400, Sigma, R5403); polyclonal Rhodopsin-specific (1:200, Sigma, R9153), Recoverin-specific (1:200, Millipore AB5585); PDE6α-specific (1:200, Thermo Fisher Scientific, PA5-32974). Appropriate FITC or cy3-conjugated secondary antibodies (Jackson) were used and cell nuclei were counter stained with 4′,6-diamidino-2-phenylindole (DAPI). Images of immunostaining were obtained by computer-assisted microscopy using a Nikon inverted microscopy (Eclipse TE2000-S) and images were obtained and analyzed using NIS-Element-BR software (Version 4.20, Nikon). Percentage of positive staining was estimated by the number of positive cells divided by total number of cells. 200–300 cells captured from randomly selected field were analysis. In all studies, a minimum of three independent experiments were performed.

Retinal Immunohistochemistry

Retinal sections were washed in 0.01M PBS and blocked for 1 hour at room temperature in PBS and 0.1% Triton X-100 with 10% goat serum before overnight incubation with primary antibody at 4 °C. The following primary

antibodies were applied: recoverin-specific antibody (Rb 1:1000, Millipore, ab5585), PDE6β-specific antibody (Rb, 1:100, Abcam, ab5663), rhodopsin-specific antibody (Rb 1:1000, Abcam, ab65694) cone arrestin-specific antibody (mouse: Rb, 1:1000, Millipore, ab15282 and human: Rb, 1:1000, Source BioScience, SBS407853). Synaptophysin-specific antibody (Rb, 1:200, abcam, ab32127), GFAP-specific antibody (Rb, 1:1000 abcam, ab7260), followed by rinsing (3 × 5 min with PBS) and 2 hours incubation with 1:400 species–appropriate Alexatagged secondary antibody at room temperature (Alexa-555 and Alexa-635, Molecular Probes, Invitrogen). Sections were then rinsed in PBS, counterstained with 1:5000 Hoechst 33342 (Invitrogen) and mounted using an antifade reagent (Prolong Gold; Invitrogen).

Light microscopy

Quantitative analysis of GFP expression in transduced neural spheres and retinal sections was achieved by light microscopy imaging, using Leica DM IL inverted epifluorescence microscope. Images were obtained using identical acquisition setting and exposure time for comparable slides and were saved at a resolution of 1200x1600 pixels.

Confocal Microscopy

Retinal sections were viewed on a confocal microscope (LSM710; Zeiss, Jena, Germany). The fluorescence of Hoechst, GFP, Alexa-555 and Alexa-635 was excited using 350-nm UV, 488-nm argon, and the 543-nm HeNe lasers, as appropriate. GFP-positive cells were first located using epifluorescence illumination and then a series of XY optical sections (approximately 0.5μm thickness) were taken in succeeding stacks to give XY projection images. Image processing was performed using Image J (Version 1.47, National Institute of Health, http://rsb.info.nih.gov/ij/index.html).

Immune suppression

Animals were immune suppressed by addition of cyclosporine A (50 mg/kg/day) and 5% fruit cordial to the drinking water [7] for 2 days prior to and 3 weeks following transplantation.

Statistical analysis

Cell quantification was analyzed using two-tailed Student t tests. Optomotor response data was analysed using a paired student t test or one-way ANOVA as appropriate (Bonferroni post hoc test for multiple comparison). Light avoidance assay was compared using one-way ANOVA when including all cases. Due to low sample size, non-parametric Kruskal-Wallis test (Dunn's test for multiple comparisons) was performed to compare responses elicited by the subgroups of animals with a high number of surviving human cells. Linear regression analysis was used to correlate behaviour with numbers of surviving cells, with F-test to determine significance. The significance (p) level for all tests was set at 0.05. Statistical analyses were carried out using SPSS version 22 (IBM).

Supplementary References

1. Warren, L., et al. Feeder-free derivation of human induced pluripotent stem cells with messenger RNA. *Sci Rep*, 2012. **2**: 657.

2. Khani, S.C., et al. AAV-mediated expression targeting of rod and cone photoreceptors with a human rhodopsin kinase promoter. *Invest Ophthalmol Vis Sci,* 2007. **48**: 3954-61.

3. Davies, W.L., L.S. Carvalho, and D.M. Hunt. SPLICE: a technique for generating in vitro spliced coding sequences from genomic DNA. *Biotechniques,* 2007. **43**: 785-9.

4. Zolotukhin, S., et al. Recombinant adeno-associated virus purification using novel methods improves infectious titer and yield. *Gene Ther,* 1999. **6**:973-85.

5. Singh, M.S., et al. Reversal of end-stage retinal degeneration and restoration of visual function by photoreceptor transplantation. *Proc Natl Acad Sci U S A*, 2013. **110**:1101-6.

6. Charbel Issa, P., et al. Optimization of in vivo confocal autofluorescence imaging of the ocular fundus in mice and its application to models of human retinal degeneration. *Invest Ophthalmol Vis Sci,* 2012. **53**: 1066-75.

7. West, E.L., et al. Long-term survival of photoreceptors transplanted into the adult murine neural retina requires immune modulation. *Stem Cells,* 2010. **28**: 1997-2007.
